# Apoptotic extracellular vesicles carrying Mif regulate macrophage recruitment and compensatory proliferation in neighboring epithelial stem cells during tissue maintenance

**DOI:** 10.1371/journal.pbio.3002194

**Published:** 2024-11-04

**Authors:** Safia A. Essien, Ivanshi Ahuja, George T. Eisenhoffer

**Affiliations:** 1 Genetics and Epigenetics Graduate Program, The University of Texas MD Anderson Cancer Center UT Health Houston Graduate School of Biomedical Sciences, Houston, Texas, United States of America; 2 Department of Genetics, University of Texas MD Anderson Cancer Center, Houston, Texas, United States of America; 3 Department of Biosciences, Rice University, Houston, Texas, United States of America; The Francis Crick Institute, UNITED KINGDOM OF GREAT BRITAIN AND NORTHERN IRELAND

## Abstract

Apoptotic cells can signal to neighboring cells to stimulate proliferation and compensate for cell loss to maintain tissue homeostasis. While apoptotic cell-derived extracellular vesicles (AEVs) can transmit instructional cues to mediate communication with neighboring cells, the molecular mechanisms that induce cell division are not well understood. Here, we show that macrophage migration inhibitory factor (Mif)-containing AEVs regulate compensatory proliferation via ERK signaling in epithelial stem cells of larval zebrafish. Time-lapse imaging showed efferocytosis of AEVs from dying epithelial stem cells by healthy neighboring stem cells. Proteomic and ultrastructure analysis of purified AEVs identified Mif localization on the AEV surface. Pharmacological inhibition or genetic mutation of Mif, or its cognate receptor CD74, decreased levels of phosphorylated ERK and compensatory proliferation in the neighboring epithelial stem cells. Disruption of Mif activity also caused decreased numbers of macrophages patrolling near AEVs, while depletion of the macrophage lineage resulted in a reduced proliferative response by the epithelial stem cells. We propose that AEVs carrying Mif directly stimulate epithelial stem cell repopulation and guide macrophages to cell non-autonomously induce localized proliferation to sustain overall cell numbers during tissue maintenance.

## Abstract 2

Apoptotic cells are known to signal to neighboring cells to stimulate proliferation and compensate for cell loss, but how? This study shows that apoptotic extracellular vesicles carrying MIF directly stimulate epithelial stem cell repopulation during tissue maintenance, and guide macrophages to induce localized proliferation.

## Introduction

The ability to maintain epithelial tissue homeostasis has important implications for the health of multicellular organisms. Failure to adequately replace dead or missing cells can predispose epithelia to failed tissue maintenance, loss of barrier function [1–3], and increased susceptibility to infection [4]. Alternatively, unchecked cell growth with minimal removal of defective cells can result in hyperplasia [5], a hallmark of cancer. Studies in *Drosophila* have shown that dying cells are able to secrete mitogenic signals to neighboring cells to stimulate proliferation [6]. Similar effects were observed in Hydra through the transfer of Wnt3 from dying cells to facilitate regeneration [7]. While the link between dying cells and proliferation has long been established, what is not entirely clear is how signals are transmitted from the dying cell to neighboring cells. Recent work has proposed apoptotic bodies or apoptotic extracellular vesicles as transporters of mitogenic signals from dying cells to neighboring cells [8–10].

Cells undergoing programmed cell death fragment into membrane bound vesicles that are approximately 1 to 5 μm in diameter [11–13], called apoptotic bodies [11] or apoptotic extracellular vesicles (AEVs), to prevent the contents from spilling out into the extracellular space. Similar to other extracellular vesicles, AEVs are enriched in contents that can regulate or interact with neighboring cells. For instance, AEVs can participate in the horizontal transfer of DNA [14,15], microRNA [16], splicing factors [17], and biologically active proteins [8,18]. While apoptosis has been dogmatically regarded as an “immunologically silent” form of cell death eliciting minimal inflammation compared to necrosis [19], damage associated molecular patterns (DAMPs) such as HSP70 and HMGB1 have been observed in AEVs [20]. Yet, how the contents of AEVs may regulate the local microenvironment after cell death in living tissues remains poorly understood.

The technical challenges to perturb living epithelia in the presence of intact immune system and image subsequent changes in real time has thus far prevented a detailed characterization of how apoptosis can stimulate proliferation. Zebrafish larvae possess an experimentally accessible bi-layered epidermis that is similar in structure and function to those coating organ systems in mammals [21–23], providing a system to rapidly interrogate the coordination of apoptosis and proliferation. The keratinocytes in the basal layer serve as the resident stem cell population that contributes to all of the strata in the adult epidermis [24], and also express defined markers found in epithelial stem cells such as TP63 [25–27]. Our previous work showed that these basal stem cells contribute to the clearance of AEVs that stimulate their proliferation in the tail fin epidermis of zebrafish larvae [9]. Here, we performed proteomic analysis of purified AEVs in zebrafish and identified proteins associated with tissue regeneration and modulation of the immune system. We further characterized macrophage migration inhibitory factor (MIF in mammals; Mif in zebrafish) as a putative regulator of AEV-mediated signaling during epithelial tissue maintenance. MIF has been characterized as a cytokine, chemokine, and molecular chaperone [28], and despite its name, plays a role in leukocyte recruitment [29–31] and proliferation and migration of epithelial cells [32]. These data suggest MIF is capable of exerting both mitogenic and immunogenic effects, yet how these are regulated during apoptosis and compensatory proliferation in vivo are not well understood.

This study investigates the role of Mif in apoptosis-induced proliferation of the basal epithelial stem cells in zebrafish larvae. We show that AEVs have Mif on their surface, which stimulates proliferation in surrounding epithelial stem cells via the up-regulation of phosphorylated ERK. Moreover, our findings indicate that apoptosis stimulates increased mobilization of macrophages, but their contribution to AEV engulfment and clearance is minimal. Our data suggest that AEVs carrying Mif stimulate macrophages to participate in compensatory proliferation in a cell non-autonomous manner. Together, these findings highlight the dynamic interplay between AEVs, neighboring epithelial stem cells, and macrophages during resolution of cell death and maintenance of overall cell numbers.

## Results

### Proteomic analysis of epithelial stem cell derived-AEVs (esAEVs) identifies proteins associated with wound healing and regeneration

**Fig 1:**
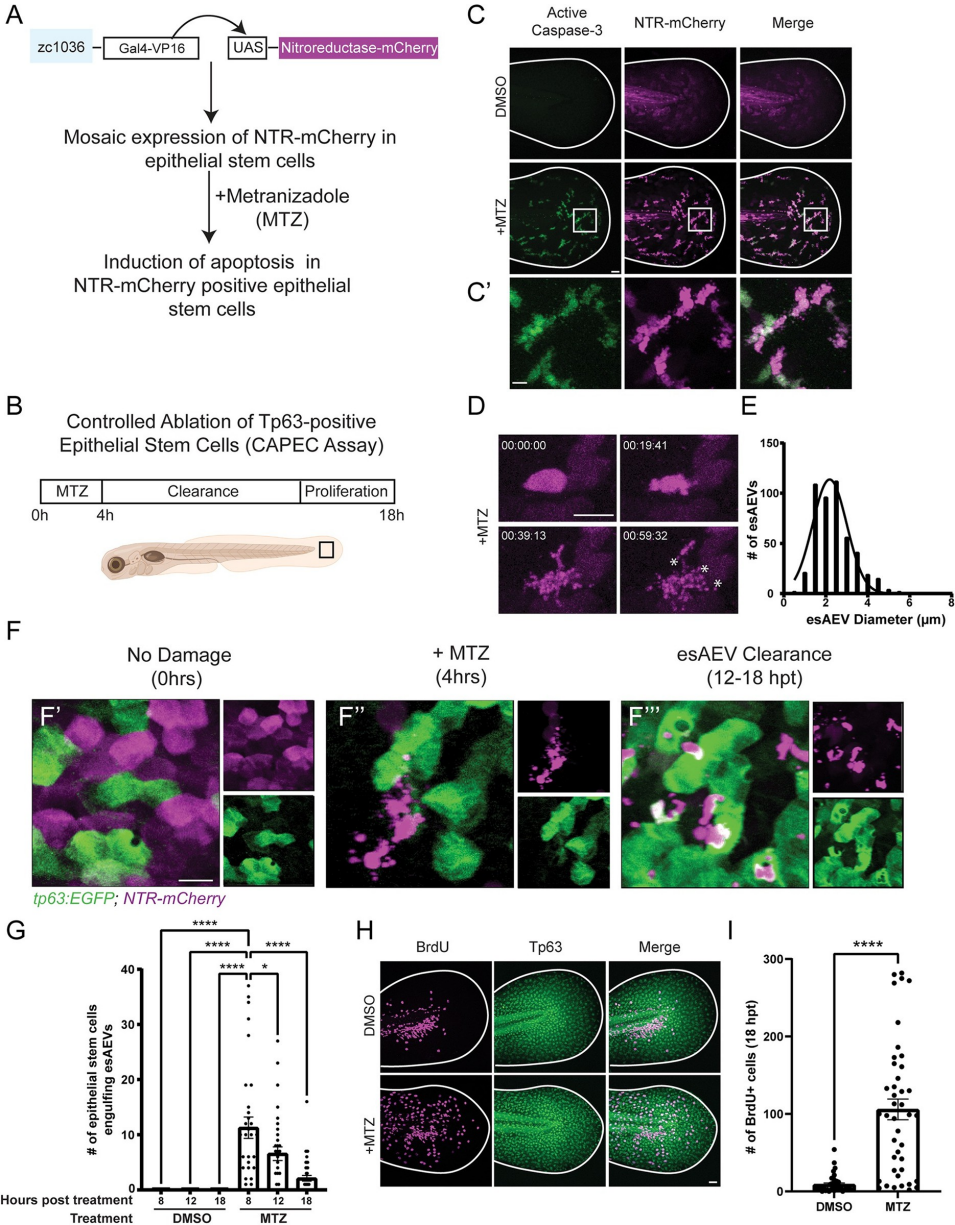
Characterization of esAEV biogenesis. (A) A description of the *Et(Gal4-VP16)*^*zc1036A*^, *Tg(UAS-1b*:*nsfB-mCherry)*^*c264*^ transgenic line. This study utilizes a Gal4/UAS zebrafish line to express the bacterial enzyme nitroreductase fused to mCherry (NTR-mCherry) in a subset of epithelial stem cells. The addition of 10 mM Metronidazole (MTZ) induces apoptosis in NTR-mCherry positive epithelial stem cells. (B) The experimental layout for the Controlled Ablation of Tp63-positive Epithelial Stem Cells (CAPEC) assay; 4 dpf zebrafish larvae are treated with MTZ for 4 h, and then washed out to enable clearance of apoptotic extracellular vesicles, and recovery of the tissue. (C and C’) Treatment with MTZ induces activated-Caspase 3 activation in NTR-positive cells. (D) After MTZ treatment, NTR-positive epithelial stem cells form AEVs over time (hh:mm:ss). (E) The diameter of individual AEVs (denoted by the asterisks in D), with average diameters of 2.413 +/− 0.927(SD) μm. (F) An overview of AEV-induced clearance and recovery. F’ depicts an undamaged tissue under homeostatic conditions. Magenta refers to NTR-positive cells, while the green denotes neighboring epithelial stem cells expressing *tp63*:*EGFP*. F” esAEV formation after 4 h of MTZ treatment. Tp63-positive cells remain unaffected by MTZ treatment. F”‘ shows the clearance of esAEVs by Tp63-positive epithelial stem cells. (G) The number of epithelial stem cells engulfing esAEVs at time points 8, 12, 18 hours post treatment. *n* = 15, DMSO 8 and 18 hpt; *n* = 18, DMSO 12 hpt. *n* = 32, MTZ 8 and 12 hpt; *n* = 39, MTZ 18 hpt. ****<0.0001, *0.0140 via a one-way ANOVA with a Tukey’s ad hoc test. (H) Representative images of the compensatory proliferation response after MTZ treatment. (I) Representation of the number of proliferating cells by measuring the amount of BrdU positive cells at 18 hpt. *n* = 38, DMSO. *n* = 42, MTZ. ****<0.0001 using a two-tailed *t* test. Scale bars: C, F, and G = 50 μm, C’ = 10 μm, D = 25 μm. The underlying data for the graphs in this figure can be found in S1 Data.

We used a zebrafish model to induce death in a subset of the basal stem cells in the bi-layered larval epidermis [21,33]. The *zc1036* Gal4 enhancer trap (BASAL-GET) line was used to drive mosaic expression of the bacterial enzyme *nsfB*, or nitroreductase (NTR) fused to mCherry [34] in basal epithelial stem cells [21] (Fig 1A). After the addition of Metronidazole (referred to as MTZ) to 4-day postfertilization (dpf) larvae for 4 h (Fig 1B), the NTR-positive cells convert MTZ into a cytotoxic byproduct that results in DNA damage [33] and apoptosis [35]. The dying epithelial stem cells display classic markers of apoptosis, such as increased activated-caspase 3 (Fig 1C), and the formation of AEVs *in vivo* (Fig 1D and S1 Movie). The dynamics of epithelial stem cell-derived AEV (esAEV) biogenesis was captured using time-lapse confocal imaging. Formation of esAEVs was observed within 1 h of cell shrinkage and had an average diameter of 2.41 microns (Fig 1E). Due to the mosaic nature of our genetic system (Fig 1F’), we can visualize the interaction of dying epithelial stem cells with the remaining healthy neighbor cells (Fig 1F”). Within 8 to 18 h posttreatment with MTZ, Tp63 positive epithelial stem cells were detected engulfing AEVs and apoptotic cell corpses (Fig 1F”‘ and S2 Movie). The most engulfment events were observed at 8 hpt (Figs 1G, S1A, and S1B), with an average of 15 individual epithelial stem cells engulfing esAEVs, and decreased over time (S1B–S1D Fig). By 18 hpt, we observed a 91.1% increase (*p* < 0.0001) in the number of actively proliferating cells that incorporated 5-bromo-2’-deoxyuridine (BrdU) (Fig 1H and 1I) compared to the DMSO only control. These findings are in line with our previous studies that demonstrated that approximately 63% of engulfing epithelial stem cells went on to divide [9] and support the mitogenic potential of esAEVs.

**Fig 2:**
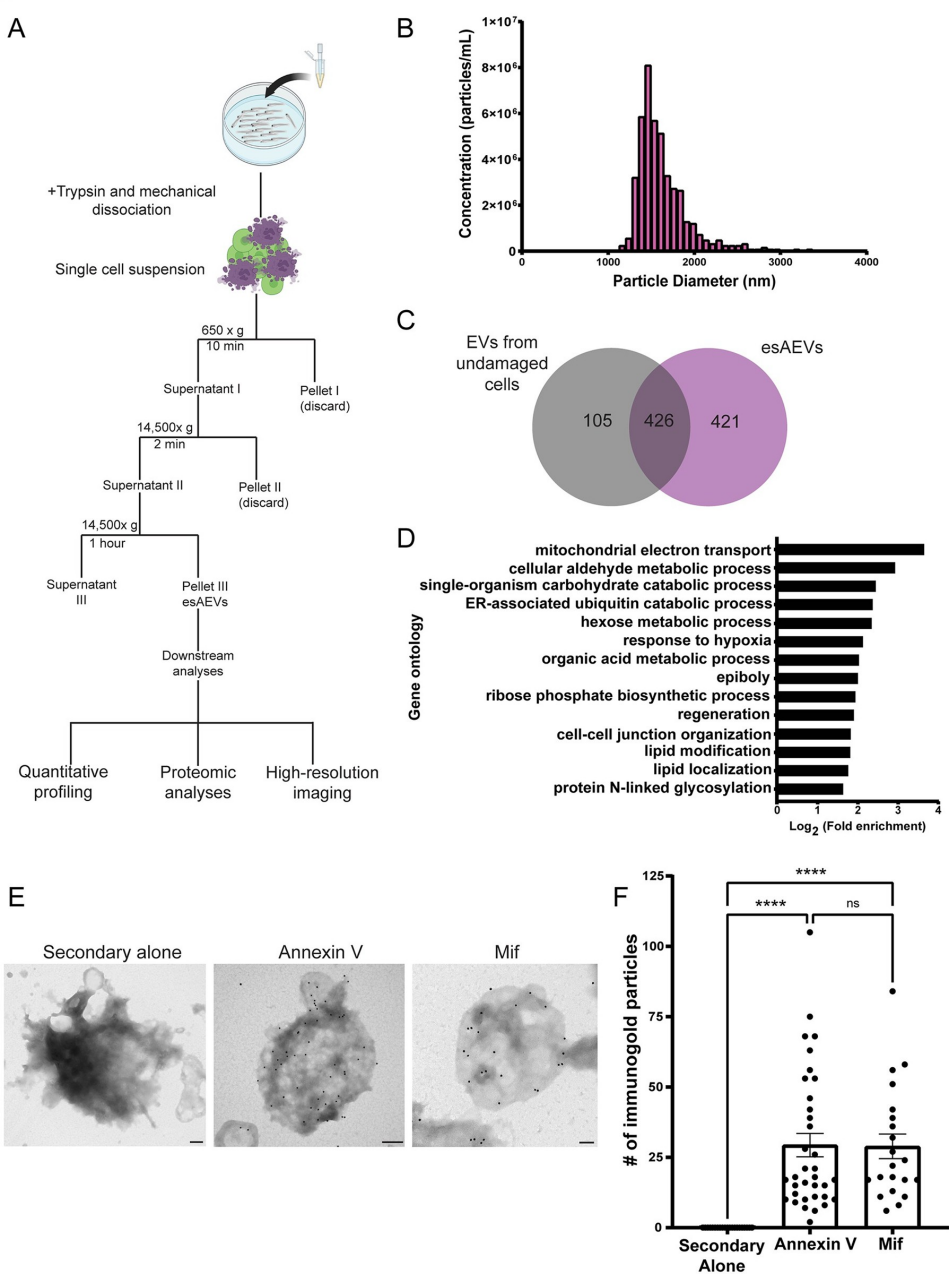
Characterization of purified esAEVs based on protein content and the localization of Mif on the surface. (A) esAEVs are isolated using differential centrifugation. For this study, isolated esAEVs were quantified for size and concentration using qNano technology, submitted for proteomic analysis, and transmission electron microscopy to validate protein localization. (B) A histogram of the size and concentration of isolated esAEVs. Minimum = 1,163 nm, Maximum = 3,338 nm, Median = 2,138 nm, and Mean diameter = 2,151 +/− 119.3 (SEM). (C) esAEVs submitted for LC-MS/MS identified a total of 925 proteins with 421 being unique to esAEVs compared to the undamaged EV control of 105. (D) Gene ontological analysis of the unique proteins found within esAEVs clustered based on biological process and log fold enrichment. (E) Electron micrographs show positive staining of Annexin V and Mif on the surface of esAEVs. (F) Shows the amount of immunogold particles per AEV. Each point represents the number of immunogold particles per esAEV. The secondary alone measures the number of immunogold particles on 21 esAEVs with a mean particle number of 0.000 +/− 0.000 (SEM). The average number of Annexin immunogold particles is 29.34 +/− 4.161 across 35 esAEVs, and Mif has an average of 28.90 +/− 4.373 across 21 esAEVs. The difference between means for Annexin V and Mif is not significant (adjusted *p*-value = 0.9998). Scale bars: Secondary alone = 200 nm, Annexin V = 200 nm, Mif = 100 nm. The underlying data for the graphs in this figure can be found in S1 Data.

To identify proteins associated with esAEVs that regulate compensatory proliferation, we purified esAEVs using differential centrifugation and performed proteomic analysis (Fig 2A). Particle size and concentration using tunable resistive pulse sensing identified a fraction enriched in approximately 2 μm AEVs (Fig 2B), consistent with that observed *in vivo* (Fig 1D and 1E). Liquid chromatography with tandem mass spectroscopy proteomic analysis of the isolated esAEVs identified 421 unique proteins when compared to extracellular vesicles isolated during homeostatic conditions with no apoptosis (Fig 2C). Gene Ontological (GO) analysis defined 14 clusters that had an enrichment score greater than or equal to 1.3, with the highest enrichment scores being pathways involved in cell metabolism (Fig 2D). This analysis also showed that esAEVs are enriched in proteins involved in biological processes such as regeneration, cell–cell junction organization, and lipid modification. We also found several major DAMPs [36–38] such as heat-shock proteins (Q90473 and Q645R1), Calreticulin (Q6DI13), S100 protein (Q6XG62), and histones H2A and H4 (Q0D272, E7FE07). We also identified proteins such as Angiosinogen (Q502R9), Pro-epidermal growth factor (EGF) (B3DH82), Low-density lipoprotein receptor-related protein (Lrp1) (A0A8M2B922), and Galectin-3 (Q6TGN4) which are known to be involved in pathways pertaining to cell proliferation or cell growth (S1 Table). Finally, we assessed the list for proteins that could play a dual role in stimulating proliferation in epithelial stem cells and regulate inflammatory responses from the immune system. This led to the identification of macrophage migration inhibitory factor (Mif) (F6PCE0). In sum, these data provide new insights into the protein components of esAEVs and their potential role in facilitating compensatory proliferation.

Mif is an attractive target for investigation due to its role in stimulating proliferation, inflammation, and immune cell dynamics. Therefore, we focused our efforts on characterizing the role of Mif in esAEV-mediated signaling. To validate the presence and localization of Mif on purified esAEVs, we assayed for Mif using immunogold labeling transmission electron microscopy. Annexin V served as a control to detect externalized phosphatidylserine (PS) on the surface of esAEVs. We observed no background staining when the gold nanoparticles were administered alone (0.000 +/−0.000). Anti-Mif nanoparticle labeling was applied, it was found to be localized on the surface of esAEVs (28.90 +/− 4.373 nanogold particles) at levels comparable to Annexin V (29.34 +/− 4.161 nanogold particles) (Fig 2E). In contrast, the Mif paralog Ddt (also referred to as Mif-like) displayed little to no detectable nanoparticle localization (4.34 +/− 3.774) (S2A and S2B Fig). Further, there was 68% (*p* = 0.0002) less Hsp70 foci, another protein identified in our mass spec data set as enriched in AEVs and commonly known to interact with protein unfolded substrates in the cytosol [39], as compared to Annexin V (S2A and S2B Fig). Additionally, we observe a 14% increase (*p* = 0.0003) in detectable Hsp70 after MTZ treatment when compared to treatment with DMSO; however, this increase is not reflected in the immunogold data (S2C and S2D Fig). We conclude that these conditions facilitate detection of proteins on the AEV surface and are sensitive enough to reveal differing levels of expression, but are insufficient to detect proteins restricted to the cytoplasm. Together, these data suggest that Mif localizes on the surface of esAEVs and could initiate Mif signaling to surrounding epithelial and immune cells.

### esAEVs transporting Mif play a role in regulating epithelial stem cell proliferation

**Fig 3:**
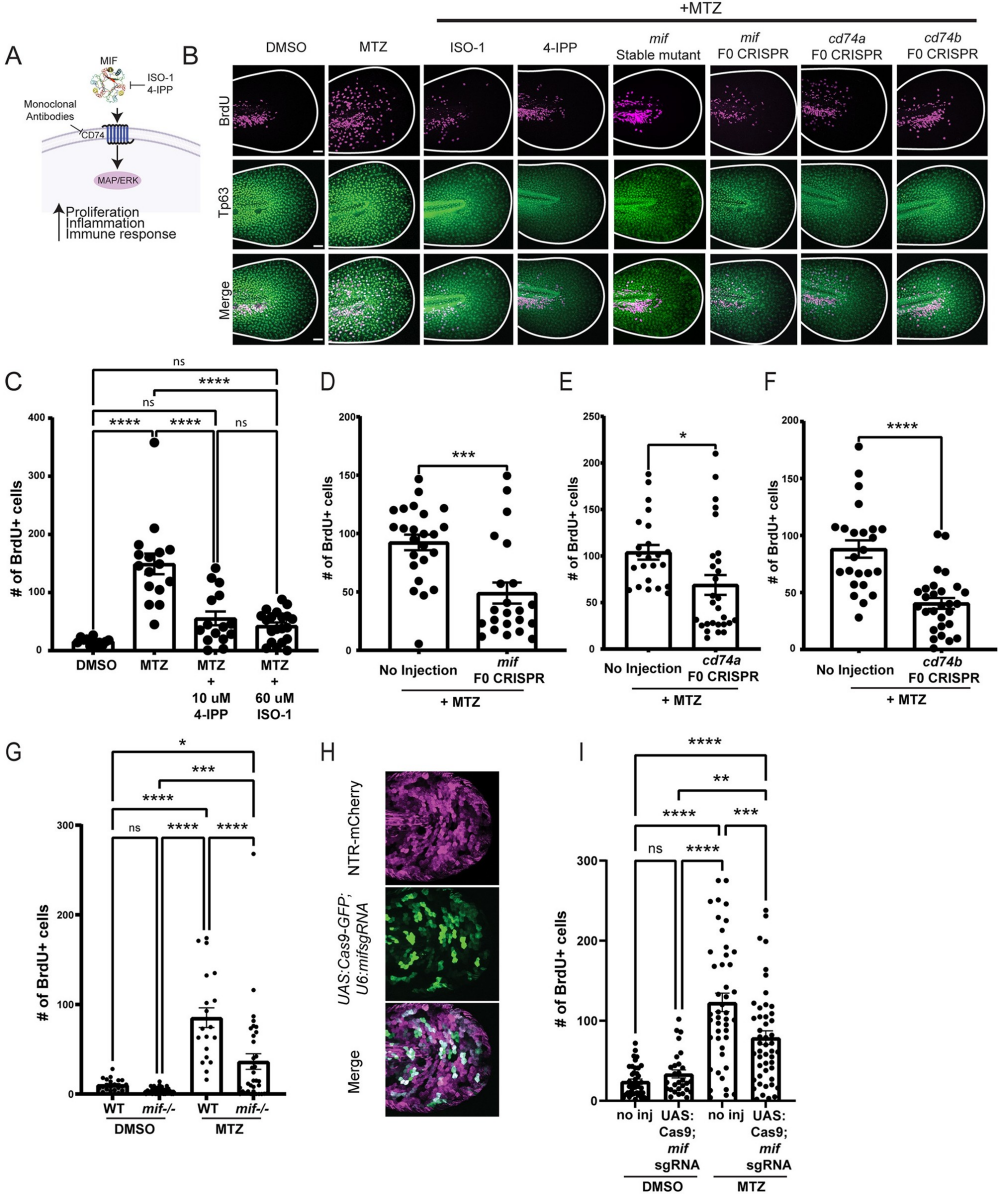
Pharmacological inhibition and genetic manipulation of Mif and its cognate receptor suppresses apoptosis-induced proliferation. (A) An overview of canonical MIF signaling upon binding to its cognate receptor, CD74. (B) Representative images of BrdU staining in Tp63-positive cells. Size bar = 50 μm. (C) The number of BrdU positive cells after Mif inhibition using ISO-1 and 4-IPP before and after apoptosis induction using MTZ. *n* = 35, DMSO. *n* = 37 MTZ. *n* = 40, MTZ + 4-IPP. *n* = 52, MTZ + ISO -1. **** < 0.0001. (D) Number of BrdU positive cells after CRISPR-mediated targeting of *mif*. *n* = 60 uninjected, *n* = 62 for *mif* CRISPR across 3 experiments ***0.0003 using an unpaired two-tailed *t* test. (E) Number of BrdU positive cells for CRISPR/Cas9 targeting of *cd74a*. *n* = 56 for uninjected, *n* = 54 for *cd74a* CRISPR. *0.0137 using an unpaired *t* test. (F) Number of BrdU positive nuclei for *cd74b* CRISPR. *n* = 62 for no injection and *n* = 61 for *cd74b* CRISPR. **** <0.001 using an unpaired two-tailed *t* test. (G) The number of BrdU positive cells for *mif* stable mutants. *n* = 53, DMSO, no injection; *n* = 60, DMSO, *mif-/-*; *n* = 44, MTZ, no injection; *n* = 65, MTZ, *mif-/-*. **** <0.0001, ** 0.0041 via a two-way ANOVA using Tukey’s ad hoc test. (H) Representative image of Cas9-GFP positive cells in NTR positive larvae. Scale bar = 50 μm. (I) The number of proliferating cells after epithelial stem cell specific deletion of *mif*. *n* = 40, no inj, DMSO; *n* = 29 *UAS*:*Cas9-T2A-GFP;mifsgRNA*, DMSO; *n* = 45, no inj, MTZ; *n* = 48, *UAS*:*Cas9-T2A-GFP;mifsgRNA*, MTZ. ** 0.0026, *** 0.0007, ****<0.0001 via a one-way ANOVA with a Tukey’s ad hoc test. All BrdU quantifications were preformed 18 h post MTZ treatment. These data are presented as means +/− SEM. Scale bar = 50 μm. The underlying data for the graphs in this figure can be found in S1 Data.

To investigate the role of Mif signaling in apoptosis-induced proliferation and esAEV signaling, we used a combination of pharmacological and genetic approaches to disrupt Mif and its cognate receptor CD74 (Fig 3A). Larvae expressing NTR were treated with DMSO (vehicle control), ISO-1 [40], or 4-IPP [41] to block Mif signaling, and then incubated with 5-bromo-2’-deoxyuridine (BrdU) to label the dividing cells. A compensatory proliferative response was observed with induced apoptosis (MTZ treatment alone compared to DMSO); however, we observed a decrease in proliferation of 63% and 67% when induced apoptosis was combined individually with either 4-IPP (*p* < 0.0001) or ISO-1 (*p* < 0.0001) treatment to inhibit Mif signaling, respectively (Fig 3B and 3C) [42,43]. The zebrafish genome contains 1 copy of *mif* [44], and 2 copies of the receptor genes, termed *cd74a* and *cd74b* [45]. To complement the pharmacological-based approach, we used the CRISPR/Cas-9 system [46] to disrupt *mif*, *cd74a*, and *cd74b*. In the F0 generation of *mif*, *cd74a*, and *cd74b* CRISPR-deleted animals (S3A–S3L Fig), or “crispants” [42,43], we observed a decrease in the number of BrdU positive epithelial stem cells for *mif* crispants (47%, *p* = 0.0003), *cd74a* crispants (35.5%, *p* = 0.0137), and *cd74b* crispants (54.5%, *p* < 0.0001) when compared to un-injected larvae treated with MTZ (Fig 3B and 3D–F). In contrast, F0 larvae injected with guide RNAs for tyrosinase (*tyr*) that disrupt pigmentation showed no statistical change in proliferation after induction of apoptosis (S4A Fig). Further, stable mutants with a 930 bp deletion in *mif* also showed a 61.6% (*p* < 0.0001) reduction in the number of proliferating cells compared to unmodified larvae treated with MTZ (Fig 3B and 3G). No differences in the number of esAEVs produced were observed in any of the experimental conditions, suggesting that perturbation of Mif does not play a role in esAEV biogenesis (S4B Fig). The CRISPR-mediated disruption to *mif* also lead to a 33% decrease (*p* < 0.0001) in the number of Tp63 positive epithelial stem cells per area after apoptosis and a slight decrease (approximately 5%) in larvae survival after induced apoptosis (S4C–S4F Fig). Together, these data support a role for the Mif/CD74 signaling axis in apoptosis-induced proliferation and restoration of overall stem cell numbers.

Our data supports the idea that Mif on the surface of AEVs plays a role in stimulating proliferation to replace cells lost by apoptosis. In addition to its presence on other EV populations [47,48], MIF has been shown to be either cytosolic or secreted [49]. To test if secreted Mif is contributing to apoptosis-induced proliferation, we first created a construct to induce overexpression human MIF fluorescently tagged with GFP (S5A Fig). Heat shock of larvae injected with *hsp70l*:*Hsa*.*MIF-GFP* resulted in a 59.3% (*p* < 0.0001) increase in fluorescent intensity and detectable MIF protein when compared to uninjected larvae (S5B–S5E Fig). After MTZ treatment in uninjected larvae, we observed a 68.3% percent increase (*p* < 0.0001) in Mif fluorescent intensity compared to undamaged larvae (S5D and S5E Fig). The increase in Mif fluorescent intensity after MTZ was not statistically different from DMSO-treated *hsp70l*:*Hsa*.*MIF-GFP* larvae, suggesting our induction strategy increased levels of MIF protein seen to those observed after damage. There was no detectable change in epithelial stem cell proliferation observed after increasing MIF, supporting the idea that apoptosis is also required for activation of Mif signaling with neighboring cells (S5F Fig).

Next, we treated larvae with Brefeldin A to disrupt proteins that are secreted through the endoplasmic reticulum (ER) and found no significant changes in esAEV formation or compensatory proliferation (S6A and S6B Fig). MIF can also be secreted via a non-classical secretion pathway that does not involve targeting to the ER [50,51] and is transported via ABC transporters [52]. Therefore, we also treated larvae with glyburide, a compound that inhibits MIF secretion in THC-1 cells by targeting ABCA1 transport [52]. Glyburide is well tolerated in larval zebrafish without causing alterations to development [53]. After treatment with 25 μm glyburide combined with MTZ, we found no significant difference in the number of esAEVs produced or number of BrdU positive epithelial stem cells (S6C and S6D Fig).

To better understand the role of *mif* specifically in epithelial stem cell derived AEVs, we used a Gal4/UAS driven approach to express fluorescently tagged Cas9 and guide RNAs [54] targeting *mif* in epithelial stem cells which would undergo apoptosis and generate AEVs after addition of MTZ (S7A Fig). We observed approximately 55% of the NTR-mCherry positive cells also expressed GFP, indicative of the percentage of cells expressing Cas9 and the *mif* guide RNAs (Figs 3H, S7B, and S7C). Using this approach, we detected a 35.8% decrease (*p* = 0.0007) in the proliferation of *UAS*:*Cas9-T2A-GFP;mifsgRNA* larvae compared to the uninjected condition (Fig 3I). Taken together, these data suggest that Mif delivery on AEVs, rather than by secretion, contributes to compensatory proliferation by the basal epithelial stem cells.

### esAEVs carrying Mif activate ERK signaling in epithelial stem cells

**Fig 4:**
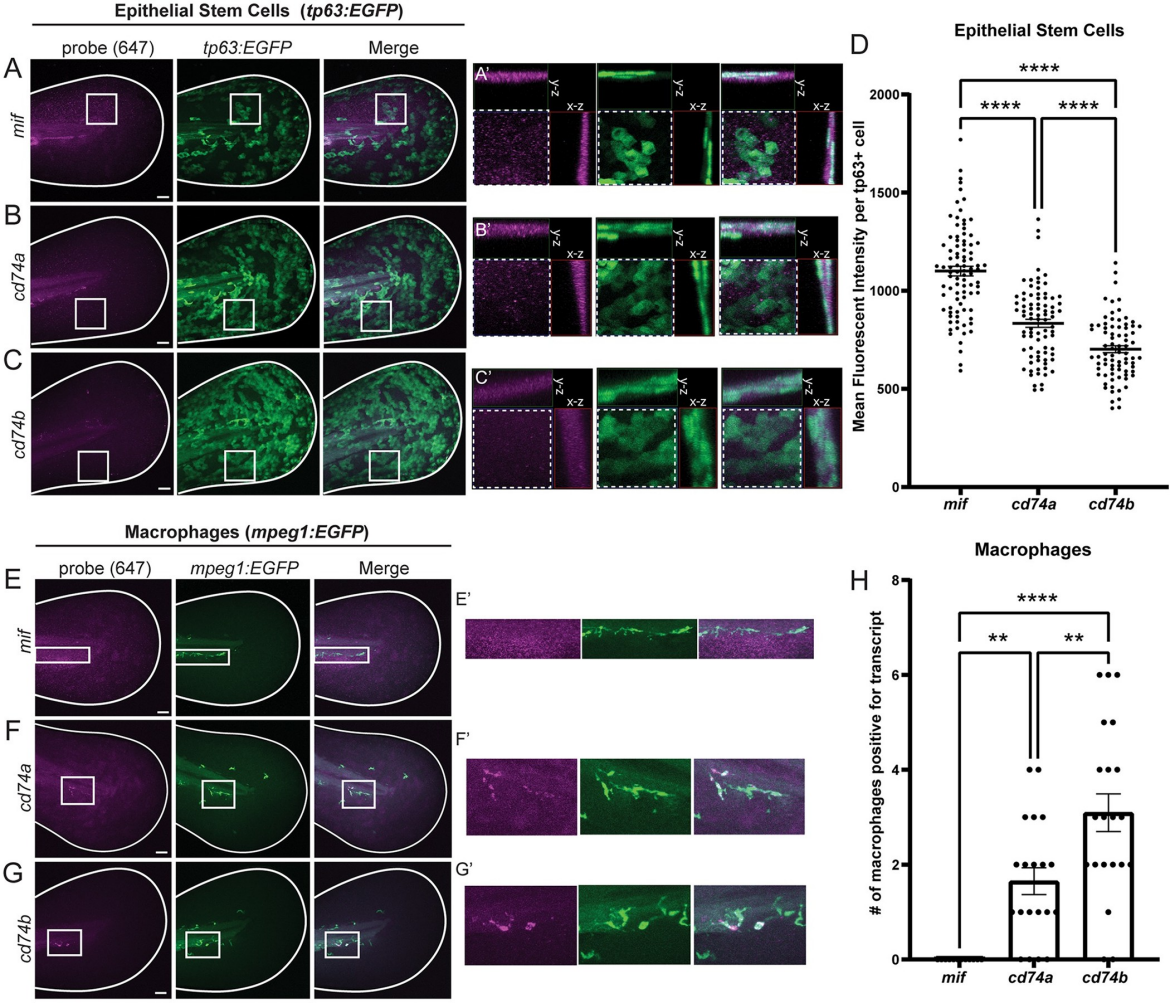
Cell type-specific expression of *mif*, *cd74a*, and *cd74b*. (A–C) Representative maximum intensity projections of *mif*, *cd74a*, and *cd74b* fluorescent *in situ* hybridization probes in the 4 dpf larvae expressing EGFP in epithelial stem cells (*tp63*:*EGFP*). (A’–C’) are ROI orthogonal projections showing the presence of *mif*, *cd74a*, and *cd74b* probe staining the same plane as epithelial stem cells. All probes were imaged in the far-red channel (647 nm). (D) Shows the mean fluorescent intensity of *mif*, *cd74a*, and *cd74b* puncta in individual epithelial stem cells. *n* = 82, *mif*. *n* = 89, *cd74a*. *n* = 79, *cd74b*. **** <0.0001 measured using a one-way ANOVA with a Tukey’s post hoc test. (E–G) Demonstrates fluorescent *in situ* hybridization probes for *mif*, *cd74a*, and *cd74b* in 4 dpf larvae expressing EGFP in macrophages *(mpeg1*:*EGFP)*. E’ shows negative localization of *mif* in macrophages. F’ and G’ shows positive staining of *cd74a* and *cd74b* in macrophages. (H) Shows the number of macrophages per larvae that are *cd74a+*, *cd74b+*, and *mif+*. *n* = 20, *cd74a*+. *n* = 21, *cd74b*+. *n* = 12, *mif*+. **0.0046 *cd74a* vs. *cd74b*. **0.0057 *cd74a* vs. *mif*. **** <0.0001 *cd74b* vs *mif*. Data are represented as mean +/− SEM. Scale bars = 50 μm. The underlying data for the graphs in this figure can be found in S1 Data.

We next sought to determine if the basal epithelial stem cells express both *mif* and the *cd74* receptors. We analyzed *mif*, *cd74a*, and *cd74b* expression using hybridization chain reaction (HCR) fluorescent *in situ* hybridization [55]. *In situ* hybridization for *mif* in *tp63*:*EGFP* transgenic animals showed high levels of expression throughout the basal epithelial cells (Fig 4A and 4D). In contrast, we observed 24% and 36% less expression of *cd74a* (*p* < 0.0001) and *cd74b* (*p* < 0.0001), respectively, when compared to *mif* transcripts within basal epithelial cells. In summary, *mif* had higher expression in basal epithelial cells, with *cd74a* showing more expression than *cd74b* (Fig 4B–4D). Immunohistochemical analyses showed Mif is distributed throughout the cytoplasm in healthy *tp63*:*EGFP* positive basal epithelial stem cells (S8A and S8B Fig), consistent with observations of MIF localization in mammalian intestinal epithelia and cultured cells [56]. Given that MIF has also been implicated in regulating immune cell function, we also examined expression within macrophages using the *mpeg1*:*EGFP* transgenic line [57]. Intriguingly, *cd74a* and *cd74b* also appear to be expressed strongly in macrophages (Fig 4E–4H). Further, *cd74a* and *cd74b* also localize to macrophages that infiltrate to sites of injury/amputation (S8C–S8E Fig). Together, this indicates that esAEVs carrying Mif can signal to CD74a or b on both epithelial stem cells and macrophages.

**Fig 5:**
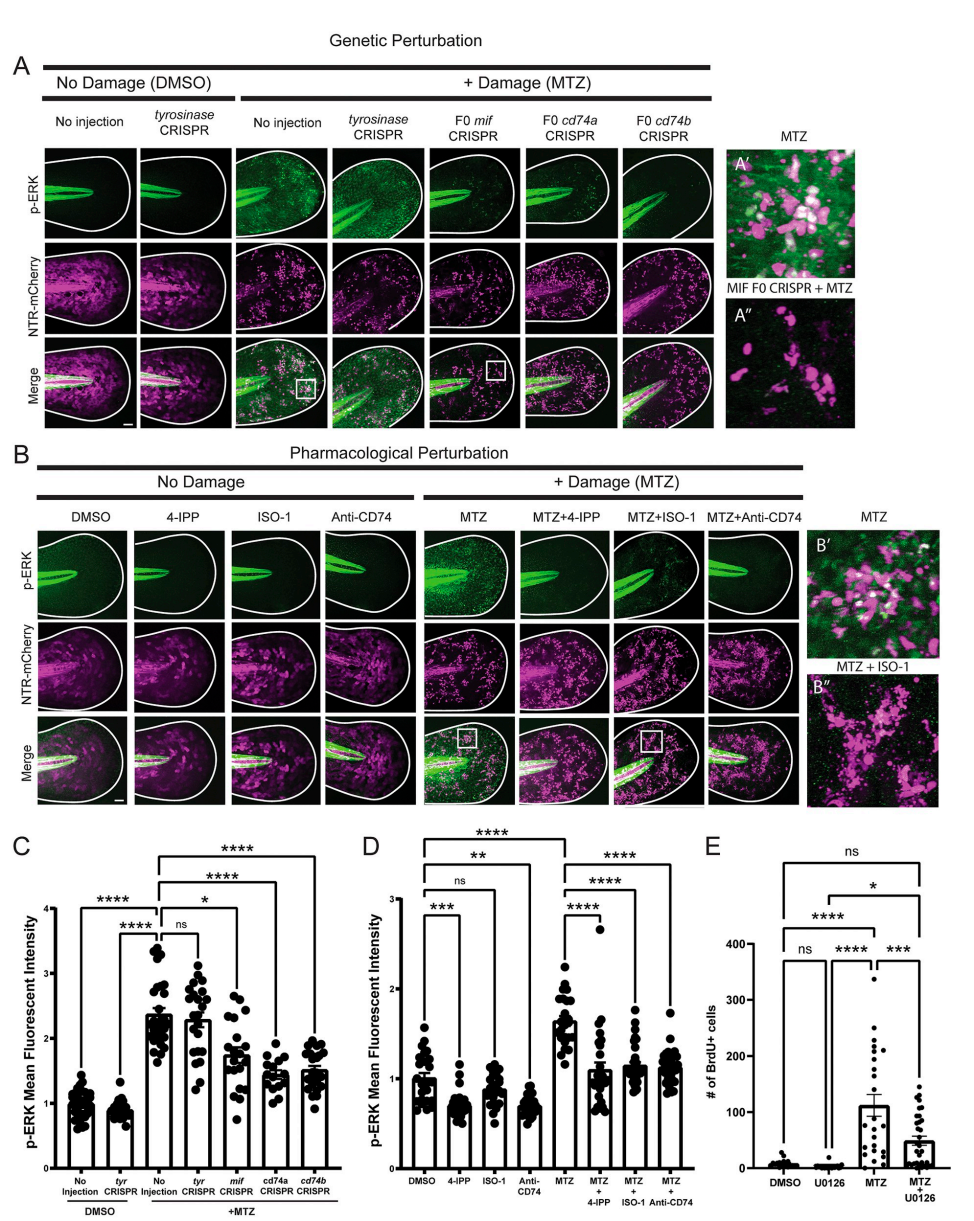
Suppression of Mif and CD74 down-regulates p-ERK signaling after apoptosis induction. **(A)** Representative images of p-ERK fluorescent intensity after CRISPR targeting of no injection, *tyrosinase (tyr)*, *mif*, *cd74a*, and *cd74b*. A’ Depicts a close up of p-ERK staining within the epithelium after MTZ treatment. A” depicts p-ERK staining in the presence of MTZ in *mif* crispants. (B) Representative images of p-ERK signaling with and without damage induction using pharmacological perturbation of Mif/CD74 signaling. B’ Depicts a close- up of p-ERK staining within the epithelium after MTZ treatment. B” depicts p-ERK staining in the presence of MTZ and ISO-1. (C) A graph showing the mean fluorescent intensity of p-ERK across CRISPR injected larvae. *n* = 70, no inj (DMSO). *n* = 53, *tyr* CRISPR (DMSO). *n* = 69 no inj (MTZ). *n* = 44, *tyr* CRISPR (MTZ). *n* = 40, *mif* CRISPR (MTZ). *n* = 39, *cd74a* CRISPR (MTZ). *n* = 40, *cd74b* CRISPR (MTZ); 2 to 3 ROIs were measured per larvae. Scale bars = 50 μm. (D) Graph depicting the mean fluorescent intensity of p-ERK across conditions of damage and no damage. DMSO, *n* = 69 ROIs. MTZ, *n* = 69. MTZ+4-IPP, *n* = 77. 4-IPP, *n* = 71. MTZ+ISO-1, *n* = 80, ISO-1, *n* = 73. Anti-CD74, *n* = 55, MTZ+Anti-CD74, *n* = 77. (E) The number of proliferating cells after damage with MEK inhibitor. *n* = 18, DMSO; *n* = 20, MEK inhibitor; *n* = 24, MTZ; *n* = 33, MTZ + U0126. Adjusted *p*-values: * 0.022, ***0.0003, ****<0.0001 via a one-way ANOVA using a Tukey’s multiple comparisons test. For each larvae, 2 to 3 ROIs within the tail epithelium were selected to measure mean fluorescent intensity. The underlying data for the graphs in this figure can be found in S1 Data.

To determine if Mif signals through CD74 to activate downstream ERK signaling [58], we analyzed levels of phosphorylated ERK (p-ERK) with and without induced apoptosis, and after disruption of Mif/CD74 signaling. Six hours post esAEV induction, we observed an average increase of 47.7% in p-ERK fluorescence after induced apoptosis when compared to the DMSO control (Fig 5A–5D). Interestingly, p-ERK fluorescence was observed adjacent to the apoptotic cells and esAEVs (Fig 5A’–5A” and 5B’–5B”), suggesting activation in healthy neighboring stem cells. Conversely, there was no observed increase in p-ERK fluorescence in macrophages at either early (6 h) or later time points (10 and 18 h) after induced apoptosis (S9A and S9B Fig). Further, p-ERK fluorescence was decreased by 20% (*p* = 0.0412) in *mif*, 36% (*p* < 0.0001), *cd74a* and 31% (*p* < 0.0001) *cd74b* crispant larvae after induced apoptosis (Fig 5A and 5C). Additionally, treatment of 4-IPP, ISO-1, or anti-CD74 combined with MTZ resulted in a decrease of 36.3% (*p* < 0.001), 31% (*p* < 0.0001), and 31.6% (*p* < 0.0001), respectively, in p-ERK fluorescence after induced apoptosis (Fig 5B and 5D). Larvae treated with an MEK inhibitor (U0126) also resulted in a 33.5% reduction (*p* < 0.0001) in p-ERK signal (S10A and S10B Fig), and a corresponding 56.5% (*p* < 0.001) decrease in the amount of proliferating cells (Fig 5E). To test whether p-ERK activity depends on the activity of dying cells, Bcl2 expression was induced in larvae [59] prior to MTZ treatment. Increased Bcl2 expression resulted in a 62% (*p* = 0.001) reduction in the number of dying cells and a 31.8% decrease (*p* < 0.0001) in the levels of p-ERK (S10C–S10F Fig), supporting the idea that dying cells play a role in stimulating p-ERK. However, these data do not rule out a possible additional role for ERK as a pro-survival signal in the neighboring cells [60–62]. Taken together, these data suggest that esAEVs carrying Mif act through interaction with CD74a/b to up-regulate p-ERK signaling to stimulate proliferation of epithelial stem cells.

### Mif plays a role in macrophage surveillance activity that contributes to compensatory proliferation in a cell non-autonomous manner

**Fig 6:**
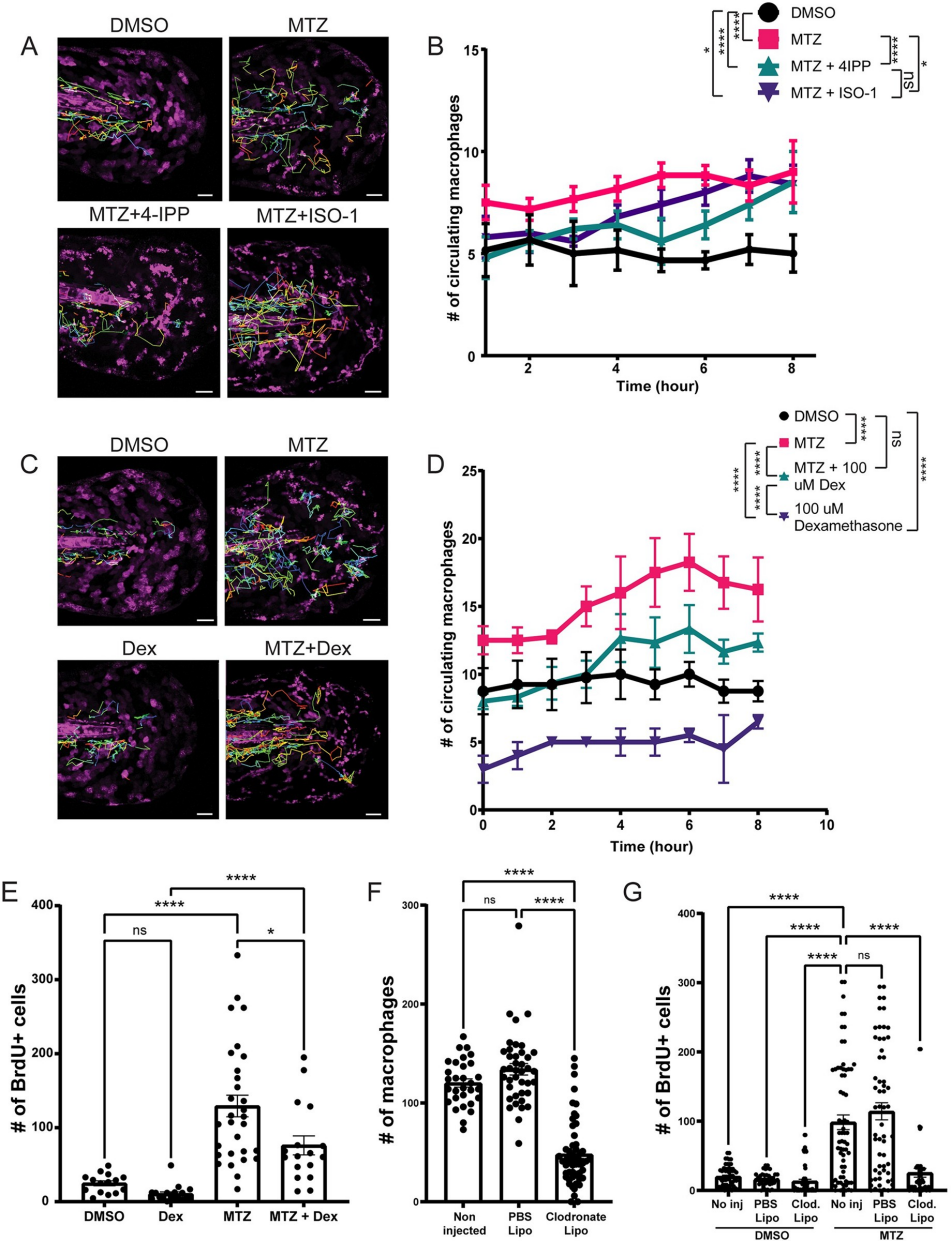
Macrophages play a role in esAEV-induced proliferation. (A) Movement tracking of macrophages over an 8-h timespan across 4 treatments denoted by the tracks. (B) Hourly quantification of macrophages extravasating out into the epithelium from the caudal vein based on pharmacological inhibition of Mif. Quantifications are averaged across multiple animals over an 8 h time period. *n* = 6 for DMSO and MTZ. *n* = 5 for MTZ+ISO-1, and MTZ+4IPP. * 0.0418, *** 0.0001, **** <0.0001. (C) Movement tracking of macrophages after treatment with Dexamethasone to suppress macrophage activity over the course of 8 h. (D) The number of macrophages surveying areas of apoptotic cells. *n* = 4 DMSO, *n* = 4 MTZ, *n* = 3 for MTZ and Dexamethasone, and *n* = 3 for Dexamethasone alone. **** <0.0001, ns = 0.1098. (E) Assessment of proliferation after treatment of dexamethasone in the presence of esAEVs at 18 hpt. *n* = 41 for DMSO, *n* = 54 for MTZ, *n* = 45 for MTZ + Dexamethasone, and *n* = 43 for Dexamethasone. * 0.012, **** <0.0001. (F) Quantifications of the number of macrophages at 4 dpf. *n* = 40, no injection. *n* = 30, control PBS liposomes; *n* = 56, clodronate liposomes. (G) The number of proliferating cells after macrophage ablation and MTZ treatment. *n* = 62, no injection, DMSO. *n* = 67, no injection, MTZ. *n* = 42, control PBS liposomes, DMSO; *n* = 60, clodronate liposomes, DMSO. *n* = 59, control PBS liposomes, MTZ; *n* = 47, clodronate liposomes, MTZ. **** <0.0001. A two-way ANOVA with a Tukey’s ad hoc test was performed to assess significance for B, D–G. Scale bars = 50 μm. The underlying data for the graphs in this figure can be found in S1 Data.

As the name implies, MIF has also been implicated in regulating the migration of macrophages [63,64]. This is in line with our observed expression of *cd74a* and *cd74b* in epithelial stem cells as well as macrophages (Figs 4A–4H and S8). To characterize the response of macrophages after induced apoptosis, we used time-lapse imaging of the *mpeg1*:*EGFP* transgenic line and observed a 43% increase (*p* < 0.0001) in macrophage surveillance activity near the apoptotic cells and AEVs in the MTZ condition compared to DMSO treated larvae (Fig 6A and 6B and S3 Movie). Interestingly, we observe a range of 3 to 10 engulfment events by the macrophages over 8 h (S11A and S11B Fig), with kinetics that could not support the complete clearance of AEVs from the tissue in this timeframe, suggesting macrophages play a role that is not solely dependent on the clearance of apoptotic cells. Treatment with either 4-IPP or ISO-1 resulted in a 23% (*p* < 0.0001) and 12.3% (*p* = 0.0484) decrease, respectively, in macrophage surveillance activity post-induction of apoptosis and AEV formation, with 4-IPP having a stronger suppressive effect than ISO-1 (Fig 6B). The observed decrease in proliferation after ISO/4-IPP treatment suggests that the macrophages may also contribute to epithelial stem cell proliferation in a cell non-autonomous manner.

To test if the presence of the immune system contributed to apoptosis-induced proliferation, we treated larvae with the anti-inflammatory dexamethasone to suppress immune cell infiltration after induced apoptosis [65]. Dexamethasone suppressed 28% (*p* < 0.0001) of the number of circulating macrophages associated with epithelial stem cell apoptosis (Figs 6C, 6D, and S11C), but had no noticeable effects on epithelial stem cell morphology under homeostatic conditions (S11D Fig). Suppression of macrophage infiltration using dexamethasone was also accompanied by a 43.5% decrease (*p* = 0.012) in the compensatory proliferation of Tp63 positive epithelial stem cells (Fig 6E). To further test this idea, we used clodronate liposomes [66] to deplete the macrophages and observed a 59.9% reduction (*p* < 0.0001) in the number of circulating macrophages (Figs 6F and S11E). The depletion of macrophages combined with MTZ treatment corresponded to a 74% reduction (*p* < 0.0001) in proliferation after induced apoptosis (Fig 6G). Additionally, we used an antisense morpholino oligonucleotide (MO) targeting the transcription factor *irf8* that has been previously shown to suppress macrophage production [67–69]. The *irf8* MO injected larvae also resulted in a 47% decrease (*p* = 0.0029) in the number of macrophages and had a 52% reduction (*p* < 0.0001) in proliferation after MTZ treatment (S11F–S11H Fig). Together, these data support the idea that the presence of macrophages contributes to compensatory epithelial stem cell proliferation after induced apoptosis and AEV formation.

## Discussion

Our studies suggest a role for AEVs derived from epithelial stem cells in stimulating compensatory proliferation after induced apoptosis by modulation of the Mif/CD74 signaling axis. These data support a growing body of research implicating AEVs as key mediators of cell-to-cell communication. For instance, apoptotic bodies have been hypothesized to transfer Wnt3 to stimulate proliferation in neighboring cells during compensatory proliferation in Hydra [7]. Our previous findings using zebrafish has established a role for AEVs carrying Wnt8a to stimulate proliferation in epithelial stem cells [9]. Further, AEVs derived from mouse mesenchymal stem cells stimulate proliferation of mesenchymal stem cells through transfer of molecules that can up-regulate the Wnt/β-catenin signaling pathway [8]. Additional effects of AEVs have been observed in stem cell populations such as endothelial progenitor cell differentiation [70], mononuclear osteoclast progenitors [18], and cardiac precursor cells [71]. Overall, these studies suggest that AEVs play an important role in regulating stem cell proliferation and tissue regeneration. Our current studies extend these findings further by providing an *in vivo* assessment of AEV activity in conjunction with an intact innate immune system.

A key question from these studies is how putative signals are transferred from apoptotic cells to neighboring healthy cells to initiate compensatory proliferation. Extracellular vesicles, including both various EV subtypes and exosomes, can transfer DNA, mRNA, and proteins to facilitate short-range communication between cells [72]. While Mif is localized throughout the cytoplasm in Tp63 positive basal epithelial stem cells under homeostatic conditions (S8A Fig), our studies suggest a critical role for surface-localized Mif on AEVs in mediating intercellular communication from dying epithelial stem cells to healthy neighboring epithelial stem cells and macrophages. So how does Mif become localized to the surface of encapsulating AEVs to initiate signaling with neighboring cells? Apoptosis disrupts the asymmetrical phospholipid distribution in the plasma membrane and exposes PS from the inner leaflet to the outer leaflet [73]. This translocation of molecules from intracellular to extracellular sites is a mechanism whereby apoptotic cells can become rapidly recognized by phagocytes [74], yet how this may also serve to facilitate signaling with neighboring cells in not well understood. We observe high levels of Mif on the surface of AEVs at the same time as PS becomes detectable. One possibility is that domains of Mif associated with the plasma membrane may also be externalized as PS becomes exposed. While there are many different phospholipid flippases and scramblases, PS exposure in apoptotic cells requires caspase-mediated cleavage of Xk-related protein 8 (Xkr8) [75,76]. Importantly, caspase activation, cell shrinkage, and DNA degradation all occurred normally in the absence of Xkr8, suggesting that the protein is not part of the apoptosis inducing machinery, but rather is specific to the process of PS exposure itself [75]. Determining which scramblases may drive this process is key to understanding how the localization of proteins such as Mif may be shuttled to the surface of extracellular vesicles during apoptosis to facilitate communication with neighboring cells.

The interaction between the cytokine MIF and its receptor CD74 can trigger downstream signaling cascades such as ERK1/2 to drive a change in cellular behaviors including proliferation, migration, and survival. In our study, we showed that AEVs promote an increase in p-ERK signaling in neighboring epithelial stem cells to drive proliferation. p-ERK activation has been shown to promote survival of cells surrounding apoptotic events [60–62] and can also serve a molecular switch to redirect fibrotic repair toward regenerative healing [77]. MIF has also been shown to drive proliferation and migration of airway muscle cells [78], spermatogonial cells [79], dendritic cells [80] in an ERK1/2 dependent fashion. While we did not observe a change in p-ERK in macrophages, other studies have shown that EVs carrying MIF can stimulate p-ERK in macrophages [81], promoting their activation and modulating their immune responses. A possible explanation for this is that CD74 can recruit additional co-receptors, such as CXCR2 [29] or CXCR4 [82], instead of CD44 [83] in an MIF-dependent manner and activate other downstream signaling events such as PI3K/Akt and calcium-dependent integrin activity [29]. What mediates the particular co-receptors that CD74 recruits is not well understood, and future characterization of these interactions may inform which downstream pathways are taking place in macrophages to facilitate compensatory proliferation.

Apoptosis has traditionally been regarded as an immunologically silent form of cell death that resolves with minimal induction of inflammation [84]. The rapid externalization of PS during apoptosis serves as an “eat-me” signal and exhibits anti-inflammatory properties [85]. These early changes in membrane components likely underlie the rapid recognition of AEVs by their neighboring basal epithelial cells, while the remaining cell corpses may produce additional signals to recruit macrophages and facilitate clearance [74]. A number of chemokines and cytokines have been shown to be involved in the recruitment of monocytes from the bloodstream [86], yet those responsible for attraction of distant macrophages to localized sites of apoptosis are not well understood. DAMPs are released during apoptosis and cellular stress events [87] and have been associated with attracting remote macrophages to sites of injury or damage [37,88]. Intriguingly, AEVs have also been implicated in the transport of DAMPs such as HMGB1 [20], a molecule involved in tissue repair [89]. Other extracellular vesicle populations such as small EVs and microvesicles can carry a variety of DAMPs [36]. While we detected only minimal amounts of Hsp70 on the surface of AEVs, HSP70 has also been shown to be localized on the surface of exosomes [90]. Exosomes derived from mesenchymal stem cells that carry MIF have also been shown to enhance myocardial repair, promote angiogenesis, and reduce fibrosis in the heart [91]. Similarly, exosome-derived MIF from nasopharyngeal carcinoma promotes metastasis by enhancing macrophage survival [48]. Alternatively, MIF can be secreted by a variety of cell types in response to tissue damage, infection, and other forms of stress, and has been shown to have pro-inflammatory and immune-regulatory effects [49,92]. We did not observe an impact on proliferation with pharmacological inhibition of Mif secretion, or with genetic overexpression of human MIF, suggesting in this context that apoptosis is critical for the ability of Mif to promote a compensatory proliferation response. Therefore, Mif may represent a novel DAMP with unique properties and functions. Together, these findings suggest that AEVs carrying Mif also play important roles in regulating diverse biological processes, including immune responses, tissue repair, and cancer progression.

Our studies indicate that macrophages contribute to AEV-mediated compensatory proliferation. We observed expression of *cd74a* and *cd74b* in macrophages and established a role for AEV-delivered Mif in guiding macrophage surveillance activity after induction of apoptosis. Importantly, dampening of inflammation via treatment with dexamethasone or depletion of the macrophage population decreased proliferation, suggesting a cell-non autonomous mechanism for the stimulation of proliferation during the re-establishment of epithelial tissue homeostasis. Macrophages have also been shown to play a key role in facilitating tissue repair [93], and that attenuation of macrophages impairs wound healing and tissue regeneration in zebrafish [94–96], salamanders [97,98], and spiny mice [99]. Our data supports the concept of regenerative inflammation, whereby timed and coordinated infiltration of immune cells interact with other cell types and alter the microenvironment via secretion of growth factors, cytokines, and lipids mediators to influence the initiation and progression of tissue repair and regeneration [100]. Apoptotic cells can prime macrophages toward a more “pro-regenerative” M2 phenotype [101], and M2 macrophages are known to secrete a variety of signals following injury [102]. MIF has been shown to induce macrophages to secrete MMP-9 downstream of ERK1/2 signaling [103], yet the types of signals macrophages secrete in response to AEV’s carrying Mif remains unclear. Identification of the signals produced by macrophages to promote compensatory proliferation of epithelial stem cells will be an interesting topic for future studies.

In summary, our studies define a role for Mif carried by AEVs in the reestablishment of tissue homeostasis in a dynamic process that engages both epithelial stem cells and macrophages. We propose that AEVs carrying Mif play a dual role in sustaining homeostatic cell numbers, to directly stimulate epithelial stem cell repopulation and guide macrophage behavior to cell non-autonomously contribute to localized proliferation. Intriguingly, we did not observe a considerable change in tissue architecture or organismal viability despite reduced stem cell numbers after induced apoptosis when Mif is absent, suggesting additional mechanisms must exist to ensure robust control of tissue homeostasis. Maintenance of stem cells and differentiated cells in the correct proportions is likely to require both feedback and feedforward signals [104]. For example, cell death or injury may modulate feedback signals that normally constrain proliferation, or conversely, dying cells can send positive signals to encourage stem cell proliferation [104]. These different levels of feedback are likely working in tandem with AEV-mediated Mif signaling to carefully control epithelial stem cell proliferation and avoid potentially pathologic cell turnover or cancer.

## Materials and methods

### Zebrafish handling and husbandry

Adult zebrafish were maintained at the MD Anderson Cancer Center fish facility in accordance with the institutional guidelines and best practices for animal care. Zebrafish embryos were maintained at 28°C in E3 medium.

### Transgenic lines

The Basal-GET line was used to drive the expression of nitroreductase in epithelial stem cells: *Et(Gal4-VP16)*^*zc1036A*^, *Tg(UAS-E1b*:*nsfB-mCherry)*^*c264*^. The Basal-GET line was crossed with a *Tg(mpeg1*:*EGFP)* ^*gl22*^ line [57] to visualize macrophage dynamics in the presence of apoptotic cells. The *Tg(tp63*:*EGFP)*^*utm2*^ *line* [21] was used to visualize epithelial stem cells. The Basal-GET line was crossed with a *Tg(hsp70*:*bcl2-2A-CAAX-GFP)* ^*utm5*^ line [59] to induce expression of Bcl2.

All transgenic lines were maintained in a wild-type AB background.

### Controlled ablation of Tp63-positive epithelial stem cells (CAPEC) assay

To temporally and spatially ablate Tp63-positive epithelial stem cells, we used the controlled ablation of Tp63-positive epithelial stem cells (CAPEC) assay. The Basal-GET line, which expresses nitroreductase fused to mCherry in a subset of basal cells, was treated with 10 mM metronidazole (MTZ) for 4 h. This treatment induced the production of epithelial stem cell-derived extracellular vesicles (esAEVs) in NTR-mCherry-positive cells, which were further studied through live imaging or post-recovery analysis.

To assess proliferation or recovery phenotypes, larvae were subjected to an 18-h recovery time prior to bromodeoxyuridine (BrdU) incorporation. After the recovery period, BrdU was administered to label dividing cells, allowing us to analyze proliferation and recovery phenotypes in response to stem cell ablation.

### Isolation of esAEVs and quantitative analysis

After inducing apoptosis in epithelial stem cells in 4 dpf larvae, a combination of mechanical dissociation, trypsonization, and centrifugation were used to isolate esAEVs. After a 4 to 5 h treatment with MTZ, 150–200 Basal-GET NTR positive larvae were transferred to a 1.5 ml Eppendorf tube, and washed once with tissue-culture grade 1× PBS. Once the larvae settled in the tube, 1 ml of pre-warmed 37°C Trypsin was added to the tube. A scalpel was used to chop the larvae for roughly 60 to 90 s. The larvae were then placed on a nutator to rock gently for 10 min. Chopping and placement on the nutator was repeated twice more. The larvae were then placed in a 4°C centrifuge for 10 min at 650 × g. The supernatant was transferred to a new tube and centrifuged for 2 min at 14,500 × g, 4°C to pellet large cells. The supernatant was transferred to a new tube and centrifuged for 1 h at 14,500 g, 4°C. In this study, tunable-resistive pulse sensing (qNano, Izon) was used to quantify the size and concentration of esAEVs. For the qNano, CPC 2000 calibration particles were used with an NP 2000 pore. esAEVs were diluted 1:50 in measurement electrolyte prior to measuring the size and concentration. Look to Fig 2A for a visual representation of this methodology.

### Proteomic profiling of esAEVs

Proteins were isolated from esAEVs and run on a gel. Coomassie gel pieces were washed, de-stained, and digested in-gel with 200 ng modified trypsin (sequencing grade, Promega) and Rapigest (TM, Waters Corp.) for 18 h at 37°C. In-solution samples were precipitated with 5:1 v/v of cold acetone at −20°C for 18 h, then centrifuged and the acetone was removed prior to treatment with Rapigest (100°C for 10 min) followed by addition of trypsin. Resulting peptides were extracted and analyzed by high-sensitivity LC-MS/MS on an Orbitrap Fusion mass spectrometer (Thermo Scientific, Waltham, Massachusetts, United States of America). Proteins were identified by database searching of the fragment spectra against the SwissProt (EBI) protein database using Mascot (v 2.6, Matrix Science, London, United Kingdom) and Proteome Discoverer (v 2.2, Thermo Scientific). Peptides were subject to 1% false discovery rate (FDR) using reverse-database searching.

### Gene ontology analysis of proteins unique to esAEVs

GO enrichment analysis was performed using DAVID [105]. The 421 unique proteins to esAEVs were uploaded to DAVID, where the following criteria were applied: Using the highest stringency for biological processes, a total of 72 clusters with different enrichment scores were returned. Applying an exclusion criterion of no less than 1.3 for enrichment scores, 14 clusters were appropriate for analysis. Within each cluster, terms with fold enrichments greater than 1.5 and the lowest FDR were further selected as biologically relevant.

### Transmission electron microscopy and Immunogold labeling

With the assistance of the M.D. Anderson Cancer Center Electron Microscopy Core Facility, isolated AEVs were submitted for immunogold labeling. AEVs were fixed in 2% Glutaraldehyde in 0.1 M PBS (pH 7.5). In order to visualize the membrane, esAEVs were whole-mounted. Fixed samples at an optimal concentration were placed as drops onto a 150-mesh carbon/formvar-coated nickel grids treated with poly-l-lysine and allowed to absorb to the formvar for approximately 1 h. Grids were rinsed onto drops of Millipore-filtered PBS, then were placed onto drops of a block reagent for 30 min. The grids were immediately placed onto the primary antibody at the appropriate dilution overnight at 4°C. As controls, some of the grids were not exposed to the primary antibody. The next day, all grids were rinsed with PBS then incubated onto drops of the appropriate secondary gold antibody at a 1:20 dilution for 2 h at room temperature. Grids were rinsed onto drops of PBS and then placed onto 2.5% glutaraldehyde in PBS buffer for 15 min. After rinsing in PBS, followed with a distilled water rinse, the grids were stained for contrast onto drops of Millipore-filtered 1% aqueous uranyl acetate for approximately 1 min and then allowed to dry. Samples were then examined in a JEM 1010 transmission electron microscope (JEOL, USA, Inc., Peabody, Massachusetts, USA) at an accelerating voltage of 80 Kv. Digital images were obtained using the AMT Imaging System (Advanced Microscopy Techniques Corp., Danvers, Massachusetts, USA). During the whole mount, AEVs were mildly permeabilized with saponin for surface antigen retrieval [106]. Primary antibodies were administered at the following concentrations: Annexin V (1:200), MIF (1:100), and D-DT (1:200). The secondary antibody was administered as a 1:20 dilution of gold nanoparticles.

### Pharmacological treatments

All pharmacological treatments were performed for 4 h unless otherwise specified. All drugs were washed out 3 times with E3. Metronidazole (MTZ) (Sigma, M3761) was made fresh daily as a 1 M stock in DMSO stored at room temperature and is protected from light. MTZ was added to E3 medium at a concentration of 10 mM. ISO-1 (Tocris, 4288), 4-IPP (Tocris, 3429), and Brefeldin A (Millipore-Sigma, B7651-5MG) were dissolved in DMSO and stored at 10 mM stocks at −20°C. ISO-1 was added to E3 medium at a concentration of 60 μm, 4-IPP and Brefeldin A are added at a concentration of 10 μm. Dexamethasone (Tocris, 1126) was stored at −20°C as a 100 mM stock and replaced on a monthly basis; 4 dpf larvae were treated at a concentration of 100 μm. The Human Anti-CD74 antibody (BioLegend, 326802) was stored at 4°C and added to E3 at a 1:400 dilution. The MEK inhibitor U0126 (EMD Millipore, 662005) was resuspended at a concentration of 50 μm and administered 6 h pre-MTZ treatment.

### Microscopy

A Zeiss LSM800 Laser Scanning confocal microscope was used for movie and image acquisition. Images were acquired as z-stacks using 20× for tail tips; 10× objective was used to acquire tiled region images of entire larvae and stitched together to present a full image. A Zeiss Axiozoom fluorescent microscope was used for acquisition of images for the BrdU immunohistochemical analyses. All microscopy images were processed using Zen software. Any adjustments made to brightness and contrast were applied consistently across images. The resulting images were then used for further analysis and quantification.

### Larvae fixation and fluorescent immunostaining

After treatment or recovery, larvae were fixed in 4% formaldehyde (Sigma) in 0.05% PBST. Larvae were left on gentle rocking overnight at 4 degrees. The fixative was washed out using 6 × 5 min washes in PBST 0.5%. BrdU detection was done by adding 2N Hydrochloric acid in ddH2O for 45 min under gentle rocking at room temperature. The HCL was washed out with 6 × 5 min washes in PBST 0.5%. Blocking was performed using 10% goat serum in blocking buffer for 1 to 2 h. Antibodies were diluted in blocking solution and used to stain larvae overnight in 4°C. The primary antibody was washed out with 6 × 20 min PBST 0.5% washes. Larvae were blocked 1 to 2 hours before the secondary antibody was added. Larvae were left overnight in the dark at 4 degrees. The secondary was washed out using 6 × 20 min PBST 0.5% washes. Body parts just above the cloaca were removed and discarded during mounting and 80% glycerol in PBS was used to preserve the fluorescence of larvae.

### Antibodies

1:200 Rat anti-BrdU (AbCam, ab6326), 1:200 Rabbit anti-Activated Caspase 3 (BD Biosciences, 559565), 1:200 Mouse anti-Phospho-p44/41 (ERK1/2) (Cell Signaling, Antibody #9101), 1:1,000 DAPI, 1:200 Rabbit anti-EGFP antibody (Thermo Fisher Scientific, OSE00002W and Abcam, ab6556), Rabbit anti-Annexin V (AbCam, ab14196), Rabbit anti-MIF (AbCam, ab65869), Rabbit anti-DDT (AbCam, ab115785), Mouse anti-BCL2 (AbCam, ab692).

### BrdU incorporation and detection

BrdU incorporation was performed using 10 mM BrdU, and 5% DMSO in E3 at 18 hpt for 45 min. After incorporation, animals were washed 3× with E3, left to recover for 45 min in E3 alone, and then fixed in paraformaldehyde overnight at 4°C. We detected BrdU positive cells after cell permeabilization using 2N HCL for 45 min. The staining protocol proceeded with applying monoclonal rat anti-BrdU primary antibody followed by the appropriate secondary antibody.

### Immunostaining and quantification of p-ERK signal

Four dpf Basal-GET larvae were drug treated with MTZ and different agents to perturb MIF activity for 4 h. Larvae were fixed 6 h post treatment and stained for p-ERK (1:200 dilution) (Cell Signaling, Antibody #9101). All p-ERK signal was visualized in the far-red channel as this has the least autofluorescence in the presence of apoptotic cells. The tail epithelium of the larvae were imaged with the same imaging parameters; 100 μm × 100 μm regions of interest were drawn around NTR-mCherry cells in control and damage conditions. Tails had a maximum of 3 ROIs. The mean fluorescent intensity was recorded across a z-stack within each ROI. All fluorescent intensity values were normalized according to the mean of DMSO.

### Fluorescent in situ hybridization

Probes for the in situ hybridization were purchased from Molecular Instruments. The genes and the accession numbers provided to Molecular Instruments are as follows:

MIF (NM_001043321.1), CD74a (NM_131590.1), CD74b (NM_131372.2).

*mif* was detected using a B1 amplifier while *cd74a* and *cd74b* were detected with a B3 amplifier. Larvae were stained according to manufacturer detection and staining procedures [55], with modifications outlined by Ruiz and colleagues [107]. All larvae were imaged in the exact same conditions to allow for comparisons between probes, and all imaging was performed using the far-red channel to minimize background fluorescence.

### Generation of CRISPR-edited larvae

The program CHOP-CHOP [108,109] was used to design highly specific guides directed against *mif*, *cd74a*, and *cd74b* and 1-cell stage embryos were injected with 2 guides for each gene with Cas9 protein (NEB, M0646T). Tyrosinase was an injection control to validate the efficacy of the Cas9 protein. Embryos were raised to 4 dpf for further experimentation. We validated the efficiency and accuracy of Cas9 by using Sanger sequencing, followed by TIDE [110] and ICE [111] (Synthego Corporation) analysis to compare the signal traces between edited and unedited animals at the expected cut sites. Stable mutants were generated by using the below guide sequences to create a 930 bp deletion in *mif* in the Basal-GET background. Multiple attempts to raise stable *cd74a* and *cd74b* mutants were unsuccessful, with the injected larvae not surviving past 21 days postfertilization.

### gRNA sequences

*tyrosinase*: 5′-GGACTGGAGGACTTCTGGGG**AGG**-3′

*mif* gRNA 1: 5′-TGAGCGAGCAGAGCGCACAC**GGG**-3′

*mif* gRNA 2: 5′-TGCTAAAGACTCGGTTCCGG**CGG**-3′

*cd74a* gRNA 1: 5′-TCCTGGGTCGAGGTGATGCA**AGG**-3′

*cd74a* gRNA 2: 5′-GCTGAATCAGAGACTCGTTC**TGG**-3′

*cd74b* gRNA 1: 5′-TTAACATGGGACCTCAGCCA**AGG**-3′

*cd74b* gRNA 2: 5′-GGCGGTCTCCTCGTCTCTCC**AGG**-3′

### Genotyping primers

*mif (*1462 bp):

F: CGTTCGCAGCTGTATCTCCT

R: AATTCTGCAACTGTACGCAC

*cd74a* (1269 bp):

F: AGCTTTCACTTAAATTACCTCACGA

R: AAAATCATGCAGACTTGAACACT

*cd74b (*1014 bp):

F: CACAGTGAGTTTAGGAAAATCC

R: CAAGTGAAGGGGGAGAAAATG

### Morpholino oligonucleotides

The morpholinos used in this study were obtained from GeneTools and the sequences were obtained from ZFIN (https://zfin.org/). The injection scheme for the *irf8* morpholino experiments were modeled after Madigan and colleagues [69]. A GeneTools 25 nucleotide scrambled (Random) control oligo was used as an injection and morpholino control.

*irf8* morpholino: AATGTTTCGCTTACTTTGAAAATGG

### Generation of hsp70l:Hsa.MIF-turboGFP larvae

The human cDNA construct of MIF-turboGFP was obtained from origene (RG205106). Overlap PCR was used to flank both ends of MIF cDNA with ATTB sites, and the AttB flanked MIF-turboGFP was inserted in pDONR221. The entry vector, 3′ hsp70l element, middle entry clone, and 5′ middle entry clone were combined in equimolar amounts with LR clonase overnight at room temperature to generate a transgenesis vector to be transformed and grown in carbenicillin plates. Transposase vector was linearized using NotI, and in vitro transcribed using mMessage. The *hsp70l*:*Hsa*.*MIF-turboGFP* larvae were mixed with transposase to concentrations of 7 ng/μl of vector and 50 ng of transposase mRNA into Basal-GET embryos. The hsp70 constructs were gifts from the Rosa Uribe lab. The destination vector, polyA tail, and transposase plasmid were gifts from the Kristen Kwan lab.

### Heat shock induction of MIF in larval zebrafish

Larvae were heat-shocked once at 37°C for 1 h at 6 h before MTZ treatment. Larvae expressing MIF-GFP and NTR-mCherry underwent MTZ treatment. Proliferation (via BrdU incorporation) was assessed at 5 dpf following recovery and prolonged MIF expression.

### Tissue-specific perturbation of *mif* using the *UAS*:*Cas9-T2A-GFP;U6*:*sgRNA1;U6*:*sgRNA2* plasmid

The *UAS*:*Cas9-T2A-GFP;U6*:*sgRNA1;U6*:*sgRNA2* plasmid was a gift from Filippo Del Bene (Addgene plasmid # 74009; http://n2t.net/addgene:74009; RRID:Addgene_74009). sgRNAs were flanked with BsmbI or Bsai restriction sites. sgRNAs flanked with restriction enzyme sites were inserted into digested UAS:Cas9-T2A-GFP plasmid sequentially using T4 DNA ligase (NEB, M02025). Positive integration of sgRNAs was verified using SP6 and T7 primers in sanger sequencing. Injection mixes were created as 7 ng/μl of plasmid with 50 ng of transposase.

### Clodronate liposome injections

The 2 dpf larvae in a BASAL-GET background crossed to *Tg(mpeg1*:*EGFP)* were injected with liposomes containing either clodronate or PBS. At 4 dpf, larvae underwent the CAPEC Assay. The number of macrophages was scored using an anti-GFP antibody (Abcam, ab6556) at 5 dpf. Clodronate and control liposomes were obtained from Liposoma (CP-005-005).

### Tracking and quantification of macrophage movement

Using a Basal-GET line crossed to *Tg(mpeg1*:*EGFP)*, macrophages were imaged every 5 min using a Zeiss LSM 800 confocal microscope. The number of macrophages outside the notochord and present in the epithelial tissue were manually counted every hour and graphed over time using GraphPad Prism. Imaris was used to track the overall movement of macrophages over an 8 to 10 h time period.

### Software

Zeiss Zen Blue was used to analyze the mean fluorescent intensity of p-ERK signal and fluorescent in situ hybridization. Zen blue was also used to quantify the number of BrdU positive nuclei. GraphPad Prism (version 9) was used for statistical analysis and graph generation. DAVID was used for protein Gene Ontology analysis. Imaris (version 9) was used to track the movement of macrophages in the larval zebrafish tail epithelium.

### Statistical analysis

Statistical analysis was performed using GraphPad Prism version 9 and 10. A *t* test was used to assess significance between 2 groups, and an ANOVA was used to determine the significance between 3 or more groups with a Tukey test as the ad hoc analysis. Results are reported with the standard error of the mean unless specified. A *p*-value less than 0.05 was considered statistically significant.

## Supporting information

S1 Fig Characterization of epithelial stem cell engulfment of apoptotic extracellular vesicles. (A) Two ROIs of 2 epithelial stem cells engulfing apoptotic cells. (B) The number of engulfment events over a 23-h timeframe. (C) Representative images of the levels of NTR positive cells up to 24 hpt. (D) Quantification of the number of dying cells over time after MTZ treatment. *n* = 3, 0 hpt; *n* = 4, 8 hpt; *n* = 3, 18 hpt; *n* = 4, 24 hpt. Adjusted *p*-values: ** 0.0058, 0 hpt vs. 18 hpt; ** 0.0042, 0 hpt vs. 24 hpt; * 0.0404, 8 hpt vs. 18 hpt; *0.0308, 8 hpt vs. 24 hpt via a one-way ANOVA using a Tukey’s ad hoc test. The underlying data for the graphs in this figure can be found in S2 Data.
(PDF)

S2 Fig Ddt (Mif-like) and Hsp70 are not localized to the surfaces of esAEVs. **(A)** A representative image of an esAEV administered a control stain of Secondary Alone (Scale bar = 200 nm), Hsp70 staining (Scale bar = 200 nm), Ddt staining (Scale bar = 400 nm), Annexin staining (Scale bar = 200 nm). (B) Quantitative representation of nanogold particles for Secondary Alone (mean+/−SEM = 0.000 +/− 0.000), Ddt (mean = 4.350 +/− 0.8438), Annexin V (mean = 41.64 +/− 5.524), and Hsp70 (13.19 +/− 3.370). Each dot represents an individual esAEV as represented in panels A. *n* = 27, Secondary Alone. *n* = 39, Annexin V. *n* = 20, Ddt. *n* = 16, Hsp70. The difference between means for Annexin V and Ddt was significant (adjusted *p*-value <0.0001). There was not a statistically significant difference in means between Ddt, Hsp70, and Secondary alone (*p* = 0.6939). (C) Representative images of Hsp70 antibody stains in zebrafish larvae. (D) The fluorescent intensity of Hsp70 antibody after MTZ treatment. *n* = 36, DMSO; *n* = 32, MTZ. ***0.0003 via an unpaired *t* test. The underlying data for the graphs in this figure can be found in S2 Data.
(PDF)

S3 Fig Genotyping of F0 CRISPR-edited larvae using sanger sequencing and fluorescent in situ hybridization (FISH). (A, E, I) The gene structures for *mif*, *cd74a*, and *cd74b*. The sgRNAs for each gene are represented along with the general locations within each respective gene. The green text highlights the PAM sequences. The text in the parenthesis refers to the ensembl genome browser accession numbers, and the information that was entered into CHOP-CHOP to design sgRNAs. (B, F, J) FISH staining of uninjected vs. *mif*, *cd74a*, and *cd74b* CRISPR larvae, respectively. (C, G, K) The comparison CRISPR editing for 2 cut sites for *mif*, *cd74a*, and *cd74b*. Magenta represents cutting efficiencies greater than 50%, and black represent less than 50% cutting efficiency as predicted by TIDE or ICE analysis. R-squared values represent the sequence alignment between control and CRISPR-edited samples. (D, H, L) Representative signal traces comparing control to CRISPR edited larvae upstream of the PAM site for *mif* (B), *cd74a* (F), and *cd74b* (J). Scale bars = 50 μm. The data underlying the pie charts in this figure can be found in S2 Data.
(PDF)

S4 Fig Characterization of the regenerative response after MTZ treatment. (A) Assessment of apoptosis-induced proliferation between uninjected larvae and *tyrosinase* CRISPR larvae after the addition of MTZ. A two-way ANOVA demonstrates that there is no statistical difference between the means for MTZ-treated uninjected and *tyrosinase* FO CRISPR. *n* = 41, uninjected, DMSO. *n* = 59 uninjected, MTZ. *n* = 23 *tyrosinase* CRISPR, DMSO. *n* = 40 *tyrosinase* FO CRISPR, MTZ. (B) Quantification of esAEVs produced across all conditions by 6 hpt. *n* = 36, DMSO; *n* = 24, MTZ; *n* = 31, MTZ + 4-IPP; *n* = 22, MTZ+ISO-1; *n* = 25, MTZ + Anti-CD74; *n* = 20, *tyr* FO CRISPR; *n* = 20, *mif* FO CRISPR; *n* = 17, *cd74a* FO CRISPR; *n* = 22, *cd74b* FO CRISPR. **** *p* < 0.0001 via a one-way ANOVA using a Tukey’s ad hoc test. (C) A representative image of the regions where Tp63 cells were quantified. (D) Representative images of Tp63 positive nuclei per condition. (E) Quantifications of the number of Tp63 positive nuclei in wild-type (WT) and *mif* crispr conditions at 5 dpf. 5 dpf: *n* = 66, WT, DMSO; *n* = 44, *mif* CRISPR, DMSO; *n* = 56, WT, MTZ; *n* = 54, *mif* CRISPR, MTZ. Adjusted *p*-values: ****<0.0001 via a one-way ANOVA. (F) Percent survival of larvae before and after MTZ treatment. Scale bar = 50 μm. The underlying data for the graphs in this figure can be found in S2 Data.
(PDF)

S5 Fig Heat-shock induction of MIF-GFP does not induce proliferation under homeostatic conditions. (A) Schema of the Tol2 construct used to drive human MIF-turboGFP downstream of the hsp70 promoter. Encoded within the genetic construct is a green heart marker using cmlc2:EGFP to initially pick select larvae with the construct. All constructs were co-injected with transposase mRNA. (B) Representative large-field images of a clutch of zebrafish larvae before and after heat-shock induction of MIF-GFP. (C) A 10× confocal image of the distribution of MIF-GFP in a larvae pre and post heat shock induction. (D) Representative images depicting MIF antibody staining with and without heat-shock inducible MIF, under undamaged (DMSO) and damage (MTZ) conditions. (E) The mean fluorescent intensity between DMSO and MTZ conditions in non-injected larvae and hsp70l:Hsa.MIF-GFP injected larvae. There are 3 ROIs selected per tail fin. n = 67, No injection, DMSO; n = 40, Hsp70:MIF-GFP, DMSO; n = 51, No injection, MTZ; n = 38, hsp70l:Hsa.MIF-GFP, MTZ. ****<0.0001, **0.0011, *0.0401 using one-way ANOVA. (F) A comparison of proliferation in heat-shocked and non-heat shocked larvae. n = 36, no inj. n = 32, heat shock. A student’s t test was used to assess differences in means. Scale bars: B = 100 μm, C = 500 μm, D = 50 μm.
(PDF)

S6 Fig Treatment with inhibitors of secretion do not alter apoptosis-induced proliferation. (A) Treatment with Brefeldin A does not affect AEV formation. (B) Treatment with increasing concentrations of Brefeldin A does not induce a significant reduction in proliferation. (C) esAEV formation after treatment with glyburide. (D) Apoptosis-induced proliferation in the presence of glyburide. Each condition had 32–40 larvae. Statistical significance was calculated with a two-way ANOVA with a Tukey’s post hoc test. Scales bars = 50 μm. The underlying data for the graphs in this figure can be found in S2 Data.
(PDF)

S7 Fig Tissue-specific targeting of *mif* in epithelial stem cells. (A) A map of the genetic Tol2 construct used to delete *mif* in epithelial stem cells. A U6 promoter was used to drive the expression of 2 different sgRNAs targeting *mif* in epithelial stem cells expressing Gal4. (B) A schematic of the ~1 kb deletion that occurs with CRISPR/Cas9 editing of the *mif* gene. (C) A gel comparing the PCR results between WT, *UAS*:*Cas9-T2A-GFP;mifsgRNA* and the *mif* stable mutants.
(PDF)

S8 Fig *cd74a* and *cd74b* expression in macrophages. (A) A representative image of MIF localization in healthy *tp63*:*EGFP* positive cells during homeostatic conditions. (B) Representative image of secondary alone control. (C) Macrophage location during homeostatic conditions in an *mpeg1*:*EGFP* transgenic line. (D) A representative image of *cd74a* transcripts in macrophages (D’) at and away from the amputation site. (E) A representative image of *cd74b* transcripts in macrophages (E’) at and away from the amputation site. Scale bars: A = 10 μm; C, D, and E = 50 μm; D’ and E’ = 5 μm.
(PDF)

S9 Fig p-ERK signaling was not detectable in macrophages. (A) Representative images of p-ERK signaling in an *mpeg1*:*EGFP* background across 3 different time points post-MTZ treatment. (B) Images of 3 ROIs selected per time point highlighting the p-ERK level in macrophages. Scale bar = 50 μm.
(PDF)

S10 Fig Apoptotic cells play a role in p-ERK activity. (A) Representative images of p-ERK staining before and after MEK inhibitor (U0126) administration. (B) Normalized fluorescent intensities of p-ERK with and without MEK inhibitor. Two–three ROIs per tail were selected. *n* = 77, DMSO; *n* = 80, MEK inhibitor; *n* = 83, MTZ; *n* = MTZ + MEK inhibitor. **** <0.0001 one-way ANOVA with a Tukey’s ad hoc test. (C) Representative images of BCL-2 stains before and after heatshock. (D) The number of apoptotic cells after heatshock. *n* = 15, -HS, DMSO; *n* = 16, +HS, DMSO; *n* = 13, -HS, MTZ; *n* = 16, +HS, MTZ. **0.001, ****<0.0001. (E) Representative images of p-ERK stains in *Et(Gal4-VP16)*^*zc1036A*^,*Tg(UAS-E1b*:*nsfB-mCherry)*^*c264*^;*Tg(hsp70l*:*bcl2-2A-CAAX-GFP)* larvae under conditions of damage and heatshock. (F) p-ERK fluorescent intensity with and without heatshock. *n* = 45, -HS, DMSO; *n* = 27, +HS, DMSO; *n* = 33, -HS, MTZ; *n* = 31, +HS, MTZ. **0.001, ****<0.0001. Statistics for C and D were determined using a one-way ANOVA with a Tukey’s ad hoc test. The underlying data for the graphs in this figure can be found in S2 Data.
(PDF)

S11 Fig Macrophage contribution to AEV engulfment, depletion with clodronate liposomes and *irf8* morpholino. (A)The number of epithelial stem cells that undergo apoptosis up to 3 h post treatment. *n* = 5 larvae, DMSO. *n* = 6, MTZ. **** <0.0001 using a two-tailed *t* test. (B) The number of engulfment events of macrophages in the presence of apoptotic cells across an 8-h timespan. *n* = 8 larvae, DMSO. *n* = 6, MTZ. *** 0.0003 using a two-tailed *t* test. (C) Fixed quantifications of macrophage presence at 6 hpt with the treatment of dexamethasone. *n* = 19 for DMSO, *n* = 33 for MTZ, *n* = 33 for MTZ + 100 μm Dexamethasone. ** 0.0072, **** <0.0001. (D) Representative images of Tp63 positive basal epithelial stem cells after treatment with Dexamethasone. Scale bar = 50 μm. (E) Ablation of the macrophage lineage using clodronate liposomes (scale bar = 200 μm). (F) Depletion of the macrophage population using *irf8* morpholino (scale bar = 200 μm). (G) Quantifications of the number of macrophages at 4 dpf. *n* = 72 for Control MO/Mock injected and *n* = 53 for *irf8* morpholino. ****<0.0001 via an unpaired two-tailed test. (H) The number of proliferating cells in *irf8* morpholino-injected larvae after MTZ treatment to induce apoptosis. *n* = 27 for DMSO, *n* = 41 for Control MO/Mock injected + MTZ, and *n* = 45 for *irf8* morpholino + MTZ. **** <0.0001. A two-way ANOVA with a Tukey’s ad hoc test was performed to assess significance. Scale bar = 200 μm. The underlying data for the graphs in this figure can be found in S2 Data.
(PDF)

S1 Table Proteins identified in the proteomic analysis. (XLSX)

S1 Movie The formation of esAEVs. Time-lapse imaging of NTR-mCherry cells undergoing apoptosis and forming esAEVs in vivo. Images were acquired every 5 min across a z-stack and compiled as a maximum intensity projection, 2.5 fps. Scale bar = 10 μm. Time = hh:mm:ss.
(AVI)

S2 Movie Epithelial stem cell engulfment of an apoptotic extracellular vesicle. Time-lapse imaging of a *tp63*:*EGFP* positive cell engulfing an NTR-positive esAEV. Images were acquired every 15 min across a z-stack for 12 h. 2.5 fps. Scale bar = 25 μm. Time = hh:mm:ss.
(AVI)

S3 Movie Macrophage movement with and without apoptosis. Time-lapse imaging of macrophage dynamics in an NTR-mCherry background. (Left) Macrophage movement with DMSO treatment. (Right) Macrophage movement after 4 h of MTZ treatment. Images were acquired every 5 min across a z-stack for 8 h. 5.0 fps. Scale bar = 50 μm. Time = hh:mm:ss.
(AVI)

S1 Data The underlying data for the graphs (Figs 1–6). (XLSX)

S2 Data The underlying data for the graphs (S1–S4, S6, and S10–S11 Figs). (XLSX)

S1 Raw Images Raw images. (TIF)

## Abbreviations

AEV: apoptotic extracellular vesicle
DAMP: damage associated molecular pattern
dpf: day postfertilization
ER: endoplasmic reticulum
FDR: false discovery rate
HCR: hybridization chain reaction
MIF: macrophage migration inhibitory factor
MO: morpholino oligonucleotide
NTR: nitroreductase
PS: phosphatidylserine
