# Supplementary figures and images for: Apoptotic extracellular vesicles carrying Mif regulate macrophage recruitment and compensatory proliferation in neighboring epithelial stem cells during tissue maintenance
Source: PLoS Biol. 2024 Nov 4;22(11):e3002194. doi: 10.1371/journal.pbio.3002194 (PMC11578469; doi:10.1371/journal.pbio.3002194)

# Supplemental Figure 1

A

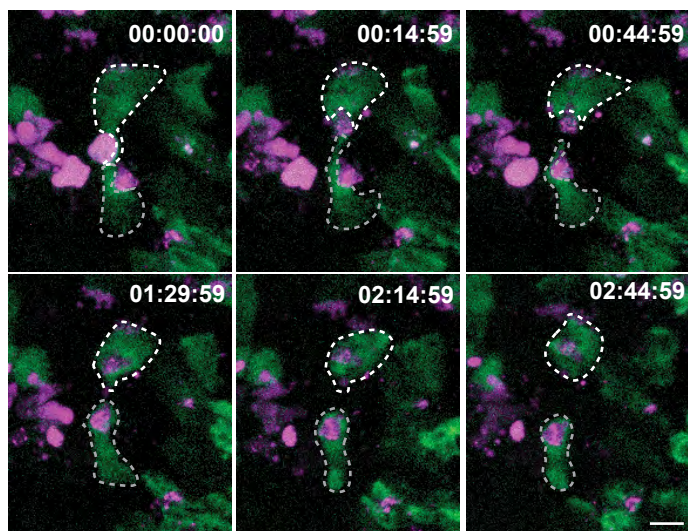

B

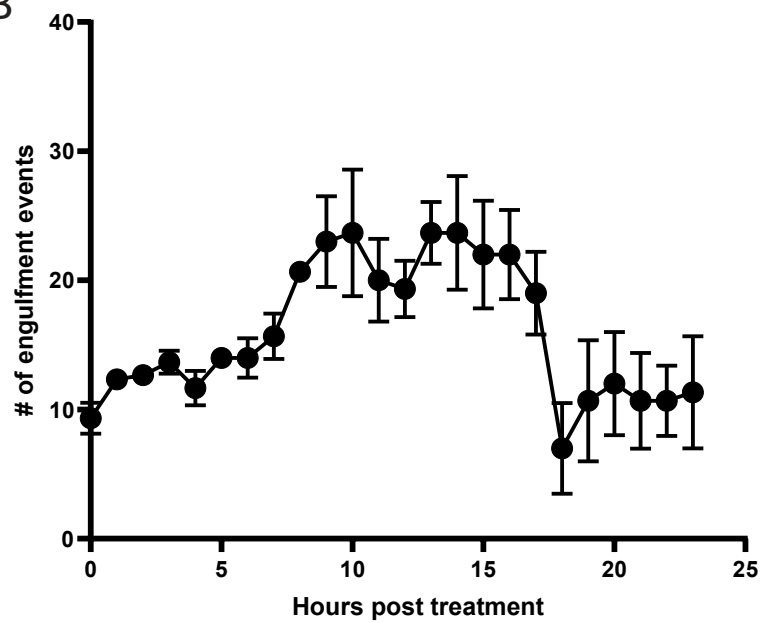

C

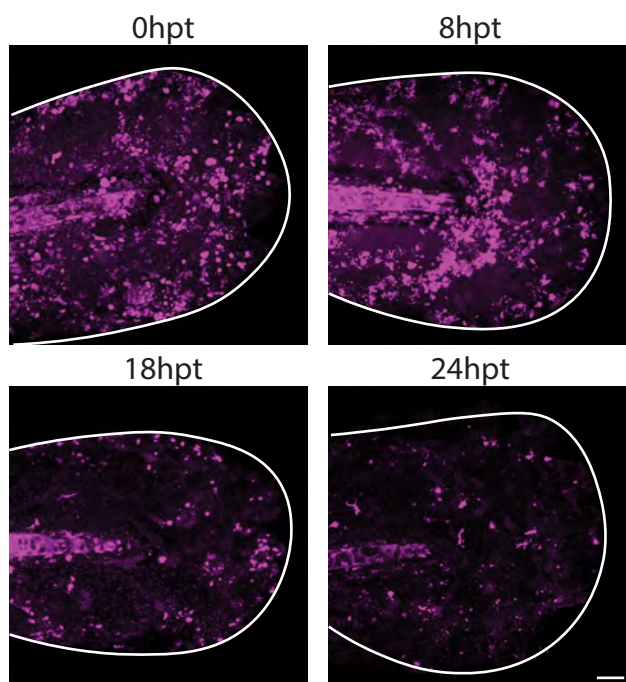

D

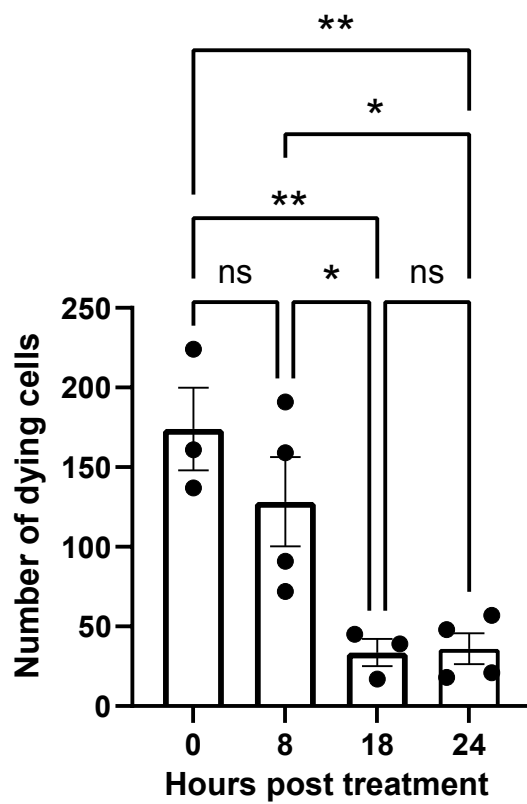

Supplement: S1 Fig — (A) Two ROIs of 2 epithelial stem cells engulfing apoptotic cells. (B) The number of engulfment events over a 23-h timeframe. (C) Representative images of the levels of NTR positive cells up to 24 hpt. (D) Quantification of the number of dying cells over time after MTZ treatment. n = 3, 0 hpt; n = 4, 8 hpt; n = 3, 18 hpt; n = 4, 24 hpt. Adjusted p-values: ** 0.0058, 0 hpt vs. 18 hpt; ** 0.0042, 0 hpt vs. 24 hpt; * 0.0404, 8 hpt vs. 18 hpt; *0.0308, 8 hpt vs. 24 hpt via a one-way ANOVA using a Tukey’s ad hoc test. The underlying data for the graphs in this figure can be found in S2 Data. (PDF) [file pbio.3002194.s001.pdf]

A

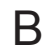

C

C'

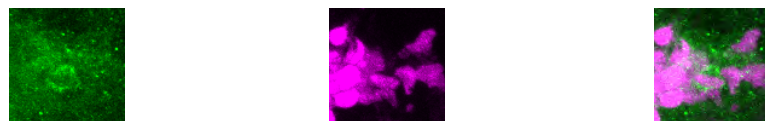

D

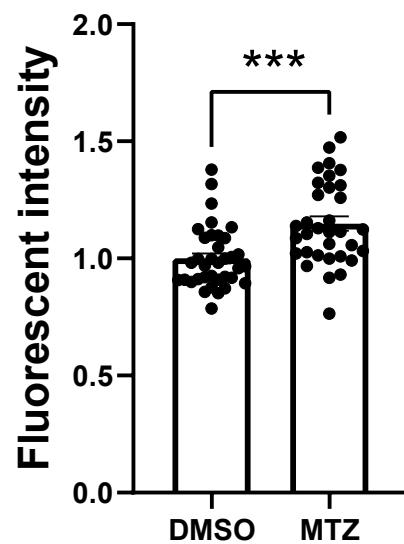

Supplement: S2 Fig — (A) A representative image of an esAEV administered a control stain of Secondary Alone (Scale bar = 200 nm), Hsp70 staining (Scale bar = 200 nm), Ddt staining (Scale bar = 400 nm), Annexin staining (Scale bar = 200 nm). (B) Quantitative representation of nanogold particles for Secondary Alone (mean+/−SEM = 0.000 +/− 0.000), Ddt (mean = 4.350 +/− 0.8438), Annexin V (mean = 41.64 +/− 5.524), and Hsp70 (13.19 +/− 3.370). Each dot represents an individual esAEV as represented in panels A. n = 27, Secondary Alone. n = 39, Annexin V. n = 20, Ddt. n = 16, Hsp70. The difference between means for Annexin V and Ddt was significant (adjusted p-value <0.0001). There was not a statistically significant difference in means between Ddt, Hsp70, and Secondary alone (p = 0.6939). (C) Representative images of Hsp70 antibody stains in zebrafish larvae. (D) The fluorescent intensity of Hsp70 antibody after MTZ treatment. n = 36, DMSO; n = 32, MTZ. ***0.0003 via an unpaired t test. The underlying data for the graphs in this figure can be found in S2 Data. (PDF) [file pbio.3002194.s002.pdf]

# Supplemental Figure 3

A

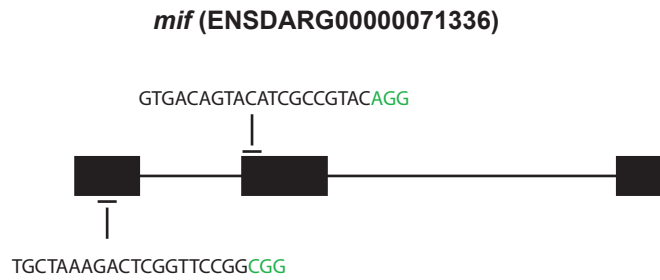

B

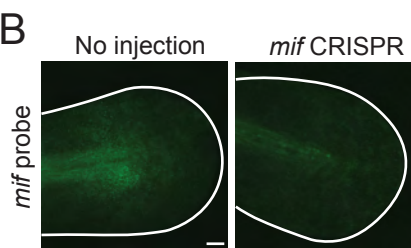

C

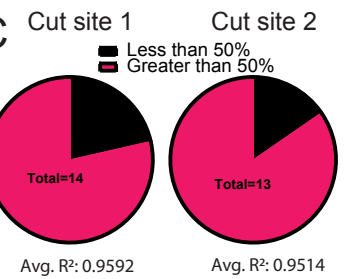

D

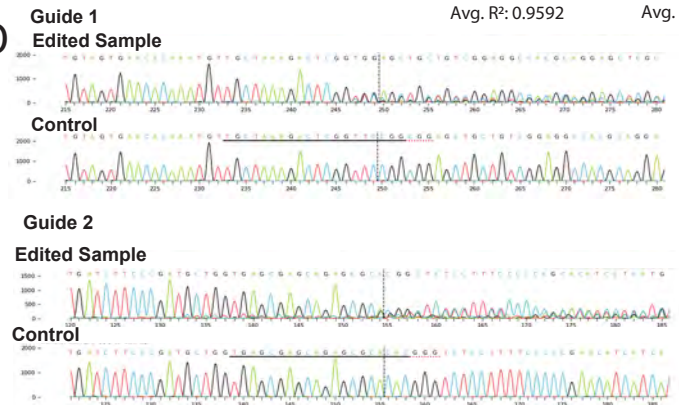

E

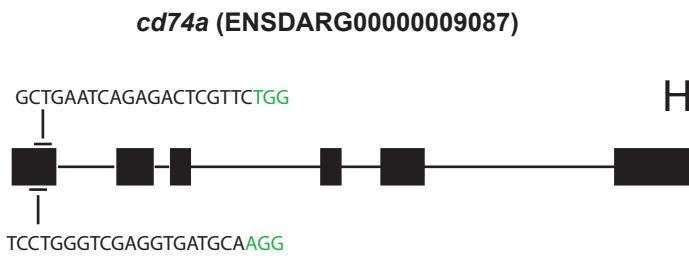

F

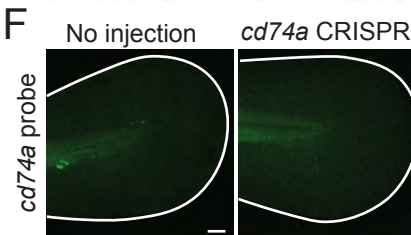

G

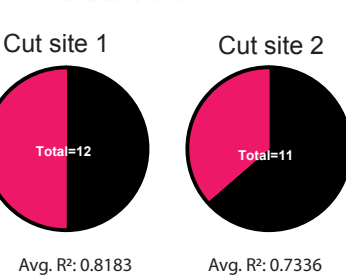

H

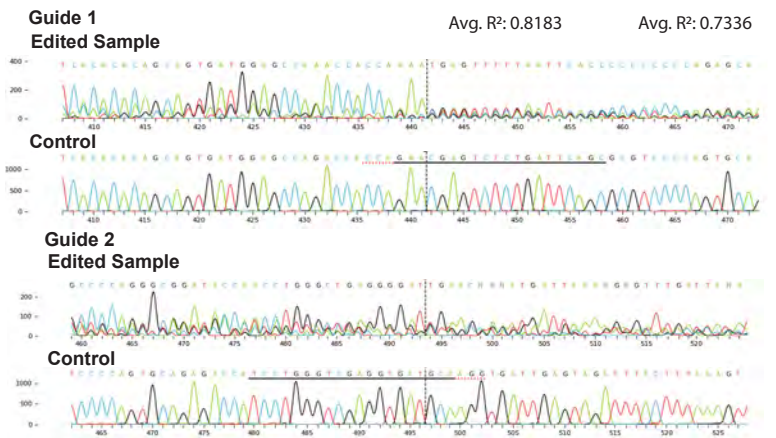

I

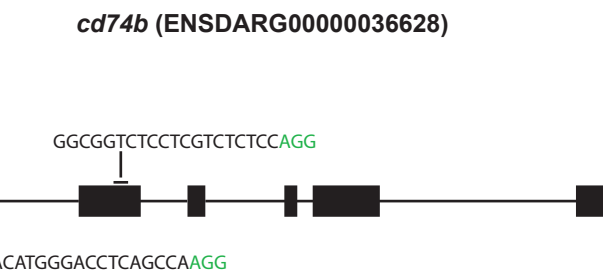

J

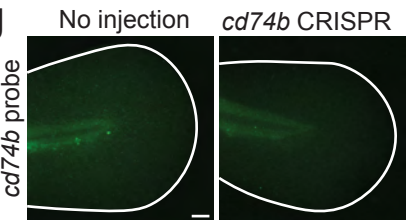

K

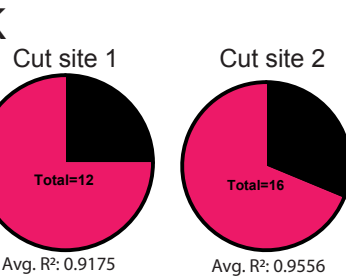

L

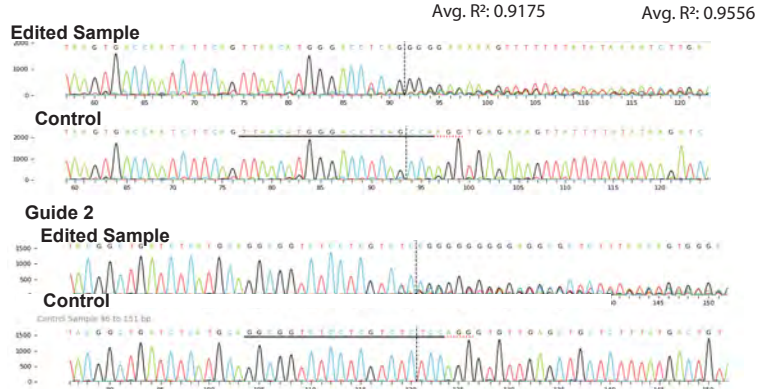

Supplement: S3 Fig — (A, E, I) The gene structures for mif, cd74a, and cd74b. The sgRNAs for each gene are represented along with the general locations within each respective gene. The green text highlights the PAM sequences. The text in the parenthesis refers to the ensembl genome browser accession numbers, and the information that was entered into CHOP-CHOP to design sgRNAs. (B, F, J) FISH staining of uninjected vs. mif, cd74a, and cd74b CRISPR larvae, respectively. (C, G, K) The comparison CRISPR editing for 2 cut sites for mif, cd74a, and cd74b. Magenta represents cutting efficiencies greater than 50%, and black represent less than 50% cutting efficiency as predicted by TIDE or ICE analysis. R-squared values represent the sequence alignment between control and CRISPR-edited samples. (D, H, L) Representative signal traces comparing control to CRISPR edited larvae upstream of the PAM site for mif (B), cd74a (F), and cd74b (J). Scale bars = 50 μm. The data underlying the pie charts in this figure can be found in S2 Data. (PDF) [file pbio.3002194.s003.pdf]

Supplemental Figure 4

A

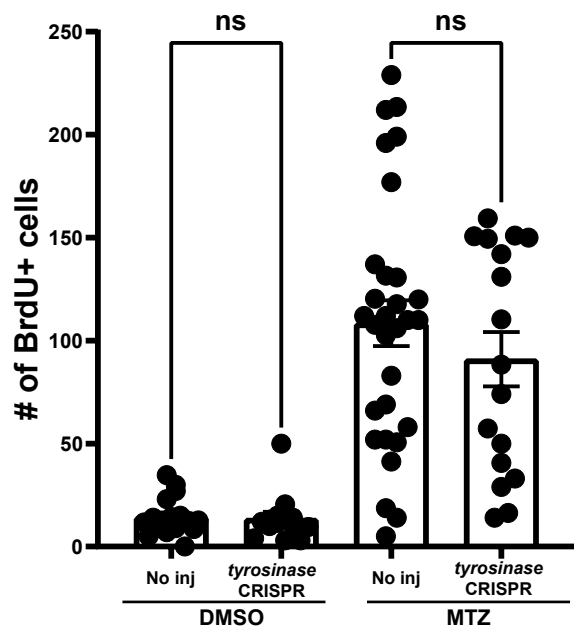

B

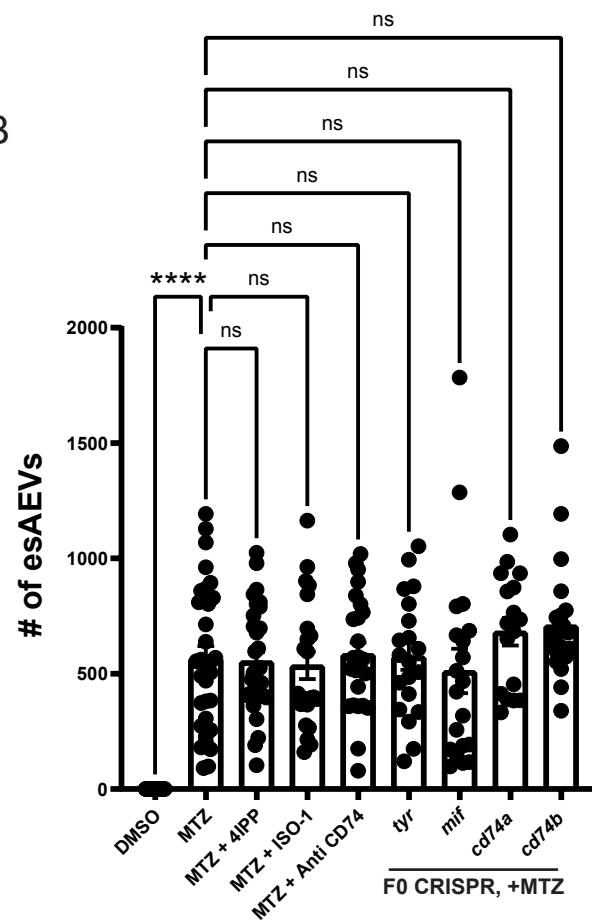

C

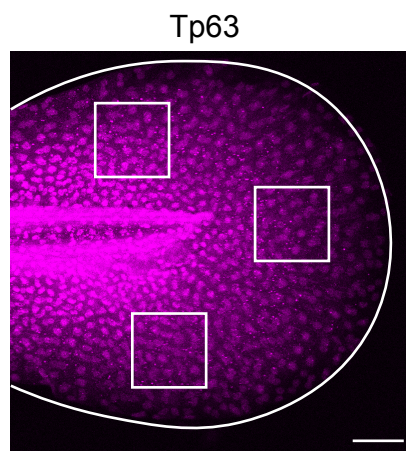

D

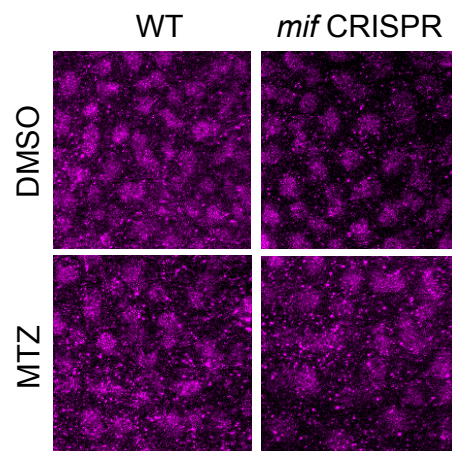

E

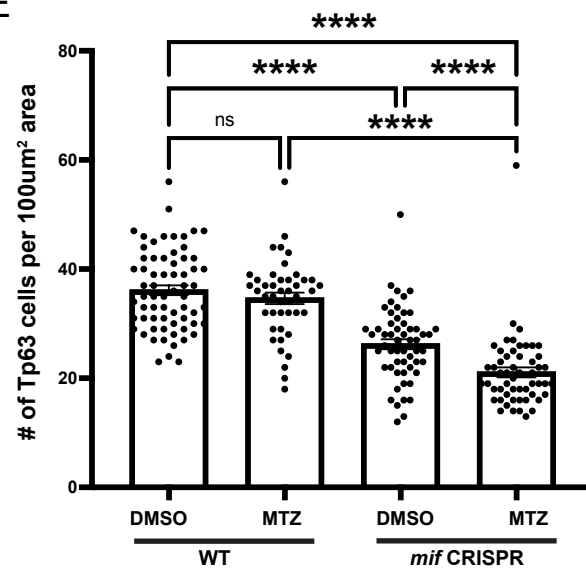

F

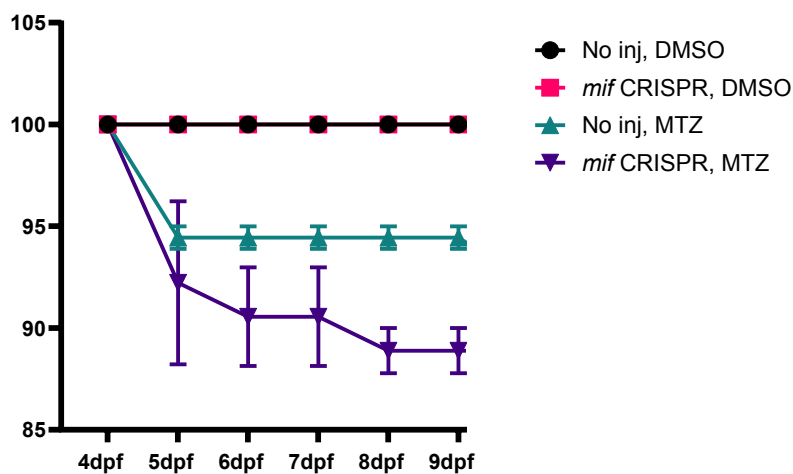

Supplement: S4 Fig — (A) Assessment of apoptosis-induced proliferation between uninjected larvae and tyrosinase CRISPR larvae after the addition of MTZ. A two-way ANOVA demonstrates that there is no statistical difference between the means for MTZ-treated uninjected and tyrosinase FO CRISPR. n = 41, uninjected, DMSO. n = 59 uninjected, MTZ. n = 23 tyrosinase CRISPR, DMSO. n = 40 tyrosinase FO CRISPR, MTZ. (B) Quantification of esAEVs produced across all conditions by 6 hpt. n = 36, DMSO; n = 24, MTZ; n = 31, MTZ + 4-IPP; n = 22, MTZ+ISO-1; n = 25, MTZ + Anti-CD74; n = 20, tyr FO CRISPR; n = 20, mif FO CRISPR; n = 17, cd74a FO CRISPR; n = 22, cd74b FO CRISPR. **** p < 0.0001 via a one-way ANOVA using a Tukey’s ad hoc test. (C) A representative image of the regions where Tp63 cells were quantified. (D) Representative images of Tp63 positive nuclei per condition. (E) Quantifications of the number of Tp63 positive nuclei in wild-type (WT) and mif crispr conditions at 5 dpf. 5 dpf: n = 66, WT, DMSO; n = 44, mif CRISPR, DMSO; n = 56, WT, MTZ; n = 54, mif CRISPR, MTZ. Adjusted p-values: ****<0.0001 via a one-way ANOVA. (F) Percent survival of larvae before and after MTZ treatment. Scale bar = 50 μm. The underlying data for the graphs in this figure can be found in S2 Data. (PDF) [file pbio.3002194.s004.pdf]

Supplemental Figure 5

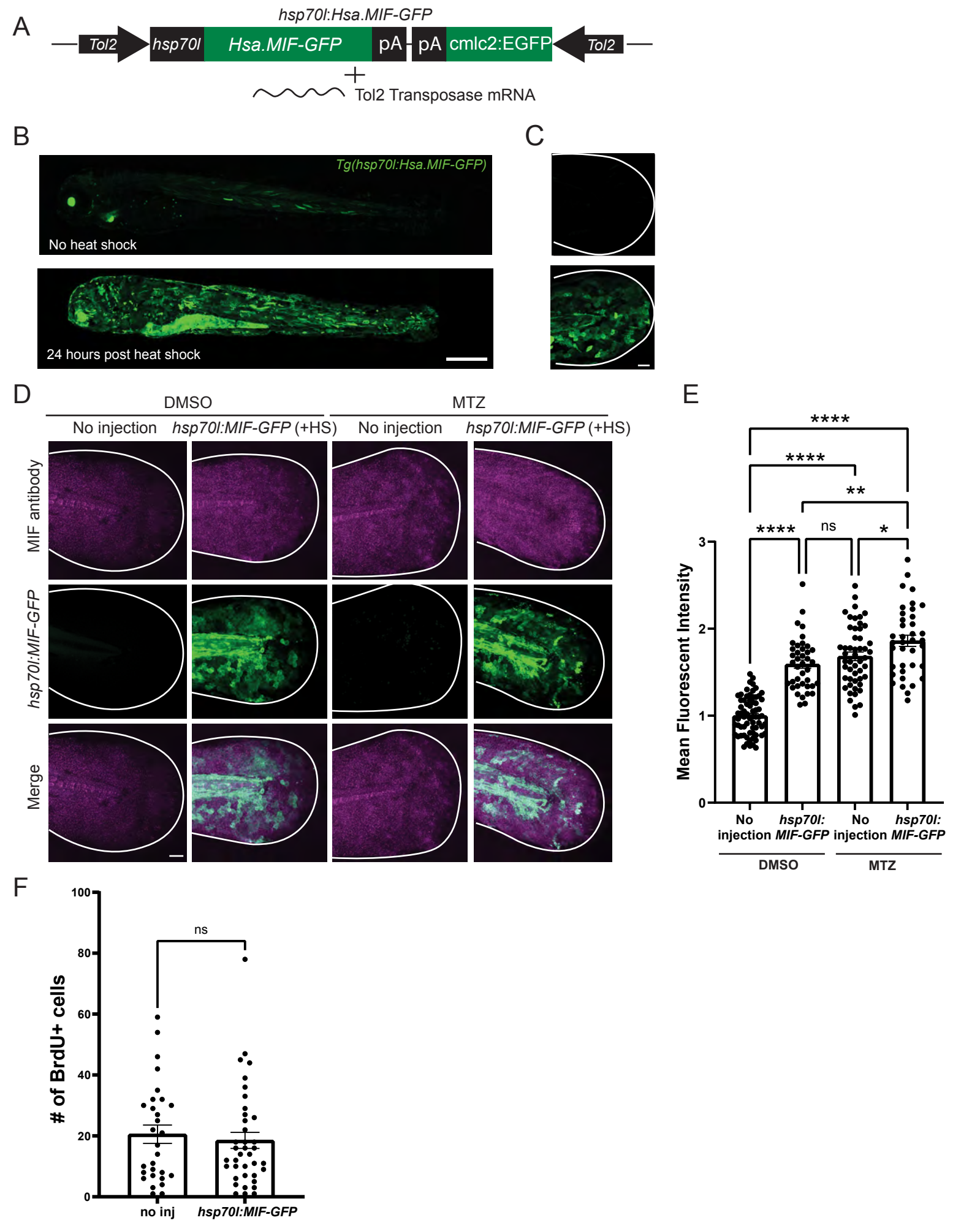

Supplement: S5 Fig — (A) Schema of the Tol2 construct used to drive human MIF-turboGFP downstream of the hsp70 promoter. Encoded within the genetic construct is a green heart marker using cmlc2:EGFP to initially pick select larvae with the construct. All constructs were co-injected with transposase mRNA. (B) Representative large-field images of a clutch of zebrafish larvae before and after heat-shock induction of MIF-GFP. (C) A 10× confocal image of the distribution of MIF-GFP in a larvae pre and post heat shock induction. (D) Representative images depicting MIF antibody staining with and without heat-shock inducible MIF, under undamaged (DMSO) and damage (MTZ) conditions. (E) The mean fluorescent intensity between DMSO and MTZ conditions in non-injected larvae and hsp70l:Hsa.MIF-GFP injected larvae. There are 3 ROIs selected per tail fin. n = 67, No injection, DMSO; n = 40, Hsp70:MIF-GFP, DMSO; n = 51, No injection, MTZ; n = 38, hsp70l:Hsa.MIF-GFP, MTZ. ****<0.0001, **0.0011, *0.0401 using one-way ANOVA. (F) A comparison of proliferation in heat-shocked and non-heat shocked larvae. n = 36, no inj. n = 32, heat shock. A student’s t test was used to assess differences in means. Scale bars: B = 100 μm, C = 500 μm, D = 50 μm. (PDF) [file pbio.3002194.s005.pdf]

Supplemental Figure 7

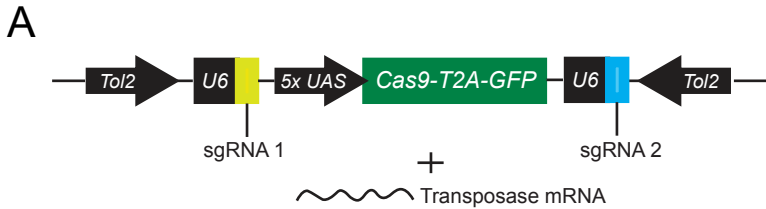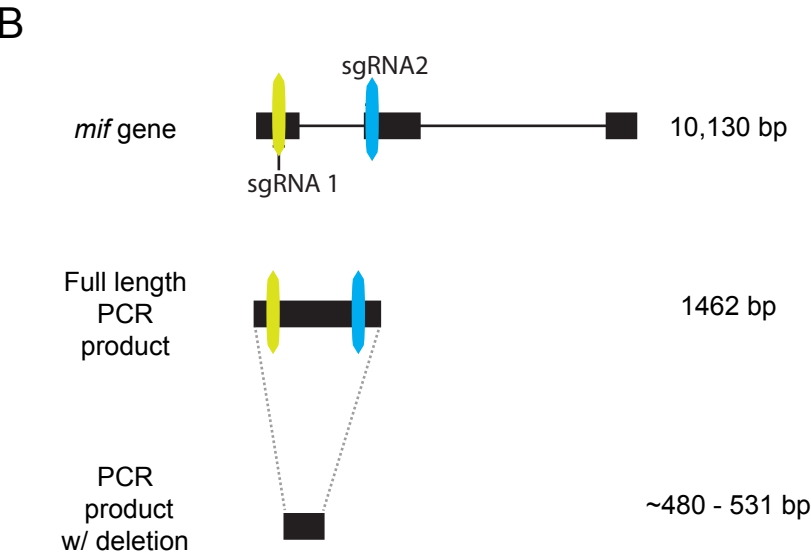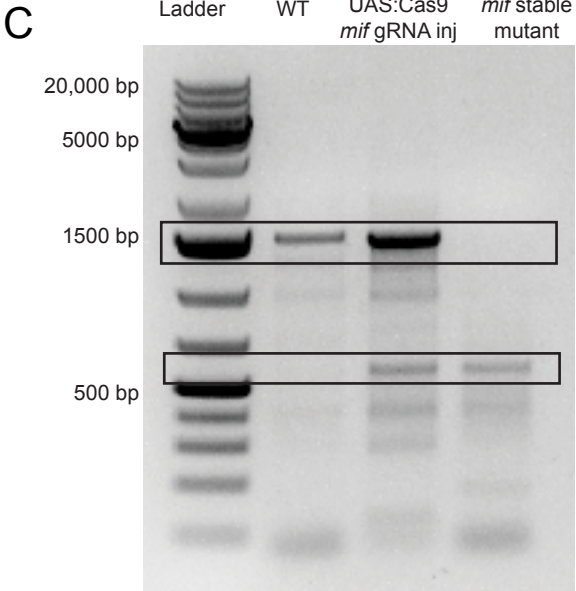

Supplement: S7 Fig — (A) A map of the genetic Tol2 construct used to delete mif in epithelial stem cells. A U6 promoter was used to drive the expression of 2 different sgRNAs targeting mif in epithelial stem cells expressing Gal4. (B) A schematic of the ~1 kb deletion that occurs with CRISPR/Cas9 editing of the mif gene. (C) A gel comparing the PCR results between WT, UAS:Cas9-T2A-GFP;mifsgRNA and the mif stable mutants. (PDF) [file pbio.3002194.s007.pdf]

Supplemental Figure 8

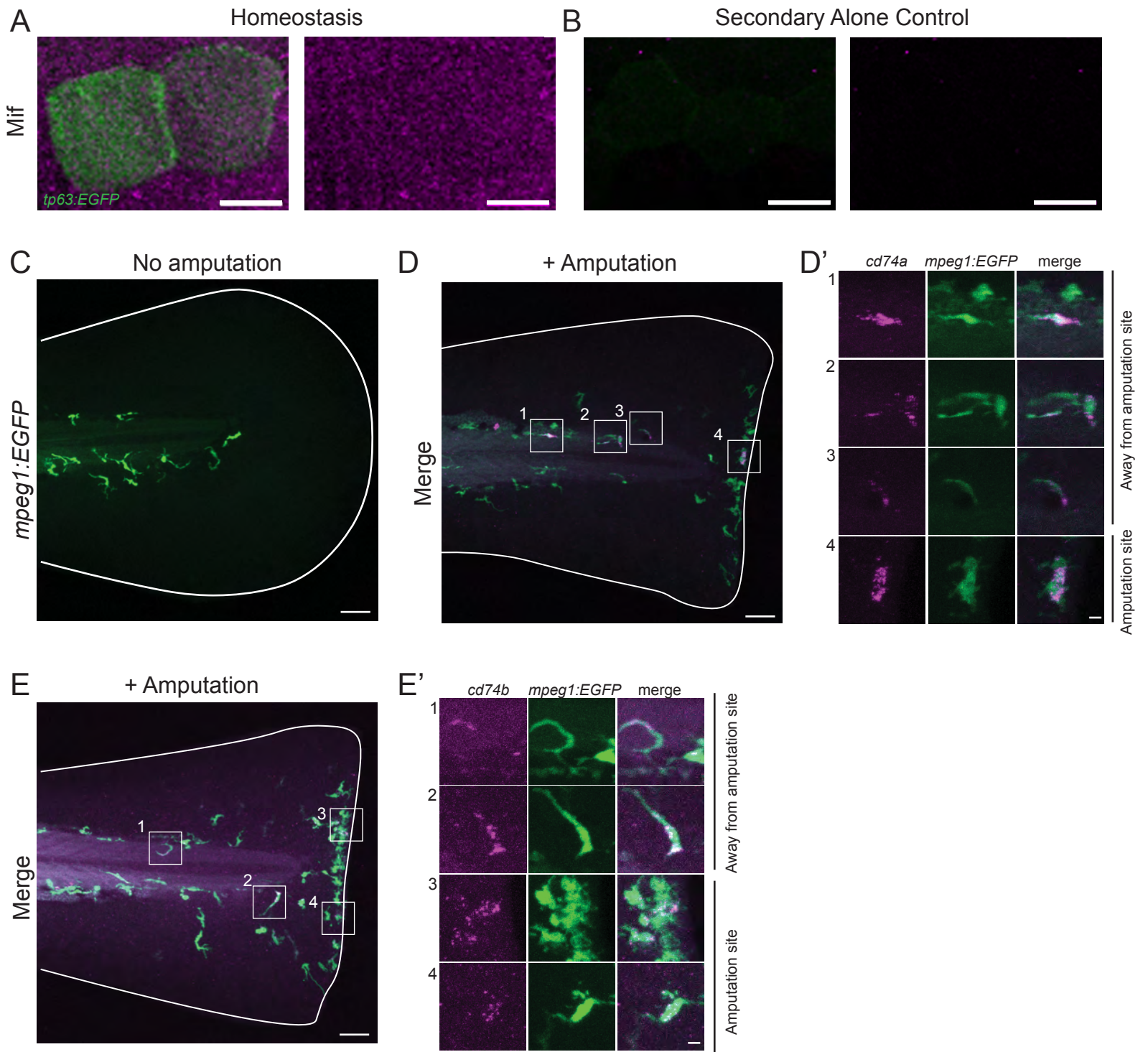

Supplement: S8 Fig — (A) A representative image of MIF localization in healthy tp63:EGFP positive cells during homeostatic conditions. (B) Representative image of secondary alone control. (C) Macrophage location during homeostatic conditions in an mpeg1:EGFP transgenic line. (D) A representative image of cd74a transcripts in macrophages (D’) at and away from the amputation site. (E) A representative image of cd74b transcripts in macrophages (E’) at and away from the amputation site. Scale bars: A = 10 μm; C, D, and E = 50 μm; D’ and E’ = 5 μm. (PDF) [file pbio.3002194.s008.pdf]

Supplemental Figure 9

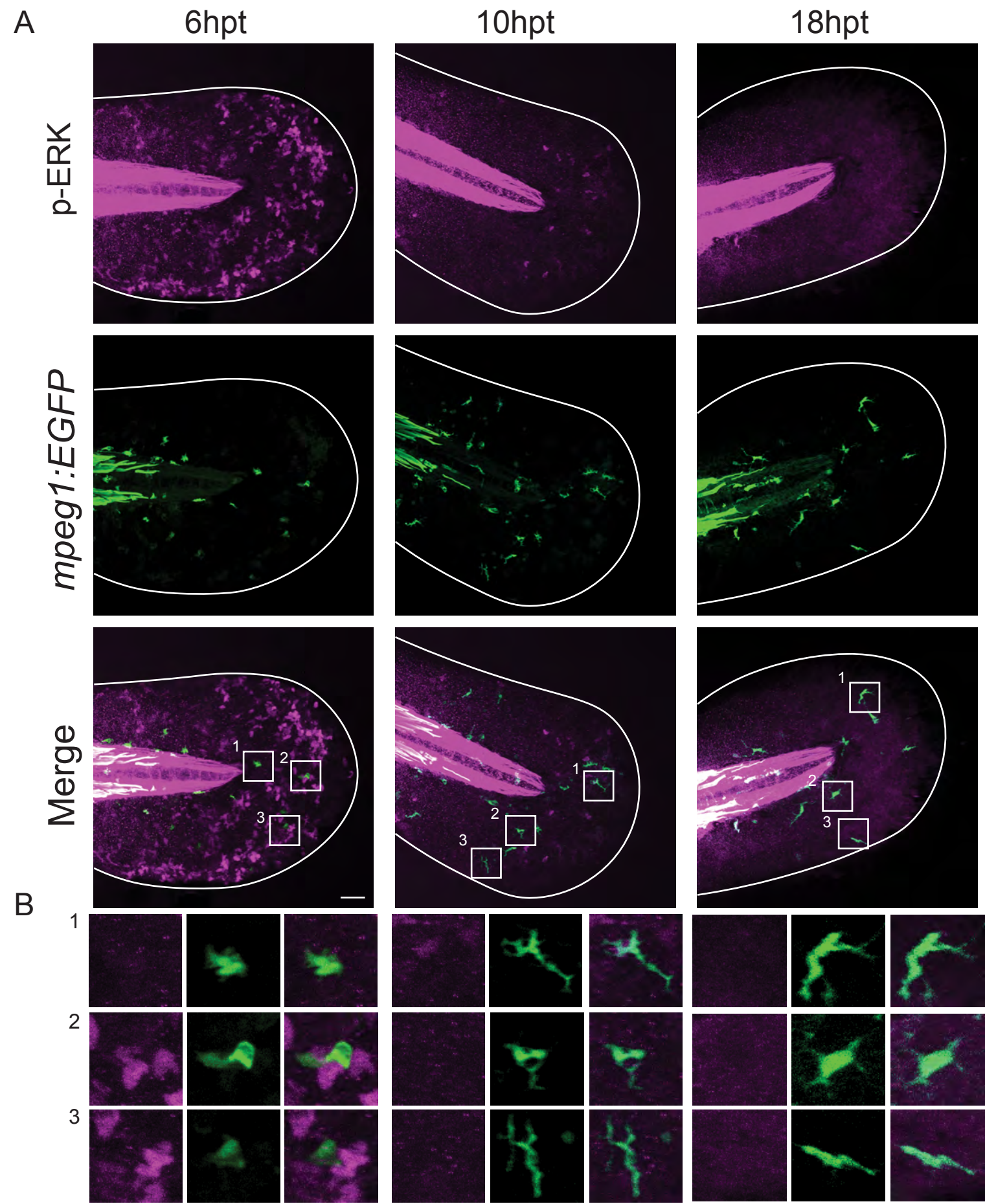

Supplement: S9 Fig — (A) Representative images of p-ERK signaling in an mpeg1:EGFP background across 3 different time points post-MTZ treatment. (B) Images of 3 ROIs selected per time point highlighting the p-ERK level in macrophages. Scale bar = 50 μm. (PDF) [file pbio.3002194.s009.pdf]

# Supplemental Figure 10

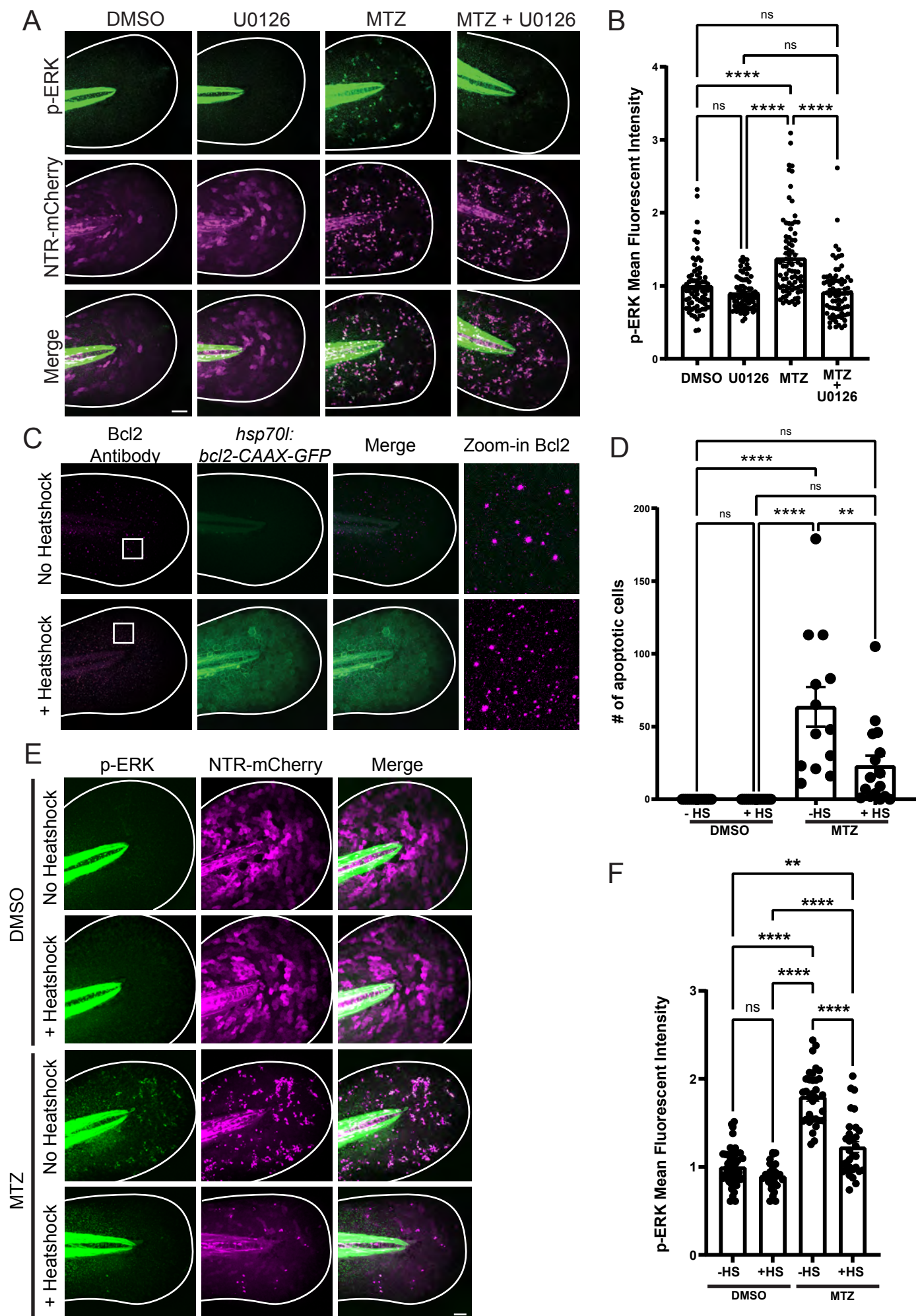

Supplement: S10 Fig — (A) Representative images of p-ERK staining before and after MEK inhibitor (U0126) administration. (B) Normalized fluorescent intensities of p-ERK with and without MEK inhibitor. Two–three ROIs per tail were selected. n = 77, DMSO; n = 80, MEK inhibitor; n = 83, MTZ; n = MTZ + MEK inhibitor. **** <0.0001 one-way ANOVA with a Tukey’s ad hoc test. (C) Representative images of BCL-2 stains before and after heatshock. (D) The number of apoptotic cells after heatshock. n = 15, -HS, DMSO; n = 16, +HS, DMSO; n = 13, -HS, MTZ; n = 16, +HS, MTZ. **0.001, ****<0.0001. (E) Representative images of p-ERK stains in Et(Gal4-VP16)zc1036A,Tg(UAS-E1b:nsfB-mCherry)c264;Tg(hsp70l:bcl2-2A-CAAX-GFP) larvae under conditions of damage and heatshock. (F) p-ERK fluorescent intensity with and without heatshock. n = 45, -HS, DMSO; n = 27, +HS, DMSO; n = 33, -HS, MTZ; n = 31, +HS, MTZ. **0.001, ****<0.0001. Statistics for C and D were determined using a one-way ANOVA with a Tukey’s ad hoc test. The underlying data for the graphs in this figure can be found in S2 Data. (PDF) [file pbio.3002194.s010.pdf]

Supplemental Figure 11

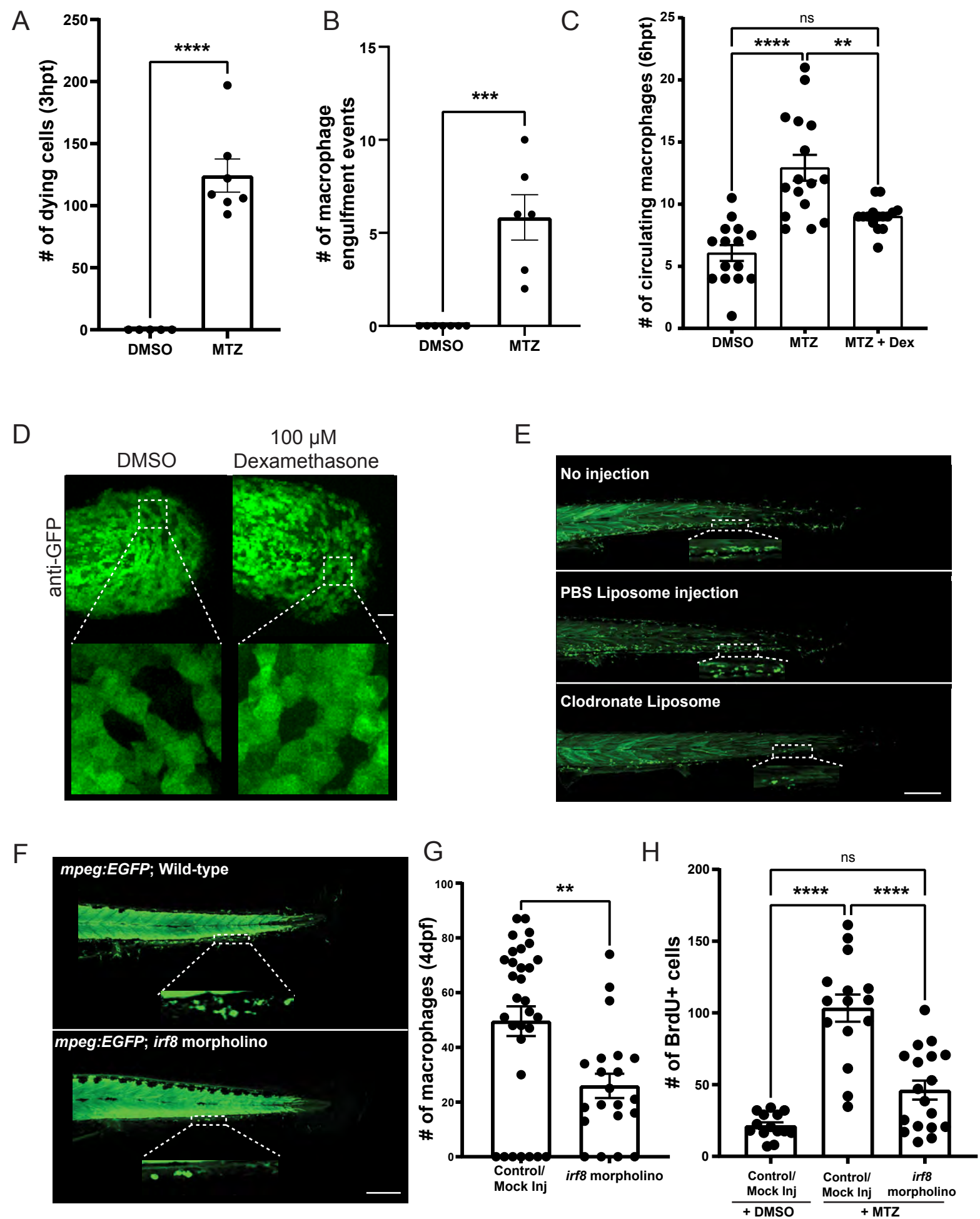

Supplement: S11 Fig — (A)The number of epithelial stem cells that undergo apoptosis up to 3 h post treatment. n = 5 larvae, DMSO. n = 6, MTZ. **** <0.0001 using a two-tailed t test. (B) The number of engulfment events of macrophages in the presence of apoptotic cells across an 8-h timespan. n = 8 larvae, DMSO. n = 6, MTZ. *** 0.0003 using a two-tailed t test. (C) Fixed quantifications of macrophage presence at 6 hpt with the treatment of dexamethasone. n = 19 for DMSO, n = 33 for MTZ, n = 33 for MTZ + 100 μm Dexamethasone. ** 0.0072, **** <0.0001. (D) Representative images of Tp63 positive basal epithelial stem cells after treatment with Dexamethasone. Scale bar = 50 μm. (E) Ablation of the macrophage lineage using clodronate liposomes (scale bar = 200 μm). (F) Depletion of the macrophage population using irf8 morpholino (scale bar = 200 μm). (G) Quantifications of the number of macrophages at 4 dpf. n = 72 for Control MO/Mock injected and n = 53 for irf8 morpholino. ****<0.0001 via an unpaired two-tailed test. (H) The number of proliferating cells in irf8 morpholino-injected larvae after MTZ treatment to induce apoptosis. n = 27 for DMSO, n = 41 for Control MO/Mock injected + MTZ, and n = 45 for irf8 morpholino + MTZ. **** <0.0001. A two-way ANOVA with a Tukey’s ad hoc test was performed to assess significance. Scale bar = 200 μm. The underlying data for the graphs in this figure can be found in S2 Data. (PDF) [file pbio.3002194.s011.pdf]

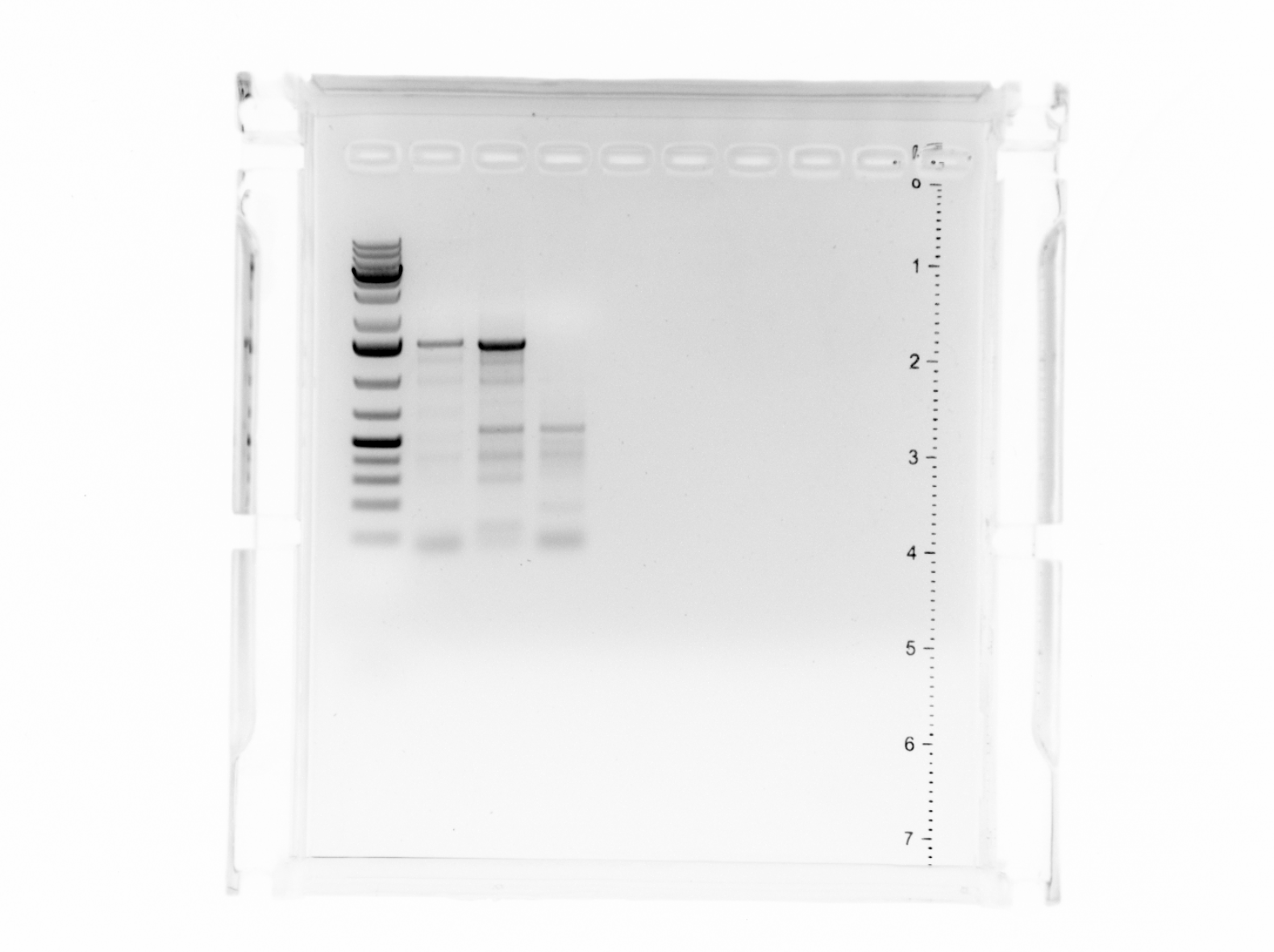

Supplement: S1 Raw Images — (TIF) [file pbio.3002194.s018.tif]
