# Supplementary material for: Apoptotic extracellular vesicles carrying Mif regulate macrophage recruitment and compensatory proliferation in neighboring epithelial stem cells during tissue maintenance
Source: PLoS Biol. 2024 Nov 4;22(11):e3002194. doi: 10.1371/journal.pbio.3002194 (PMC11578469; doi:10.1371/journal.pbio.3002194)

Supplemental Figure 6

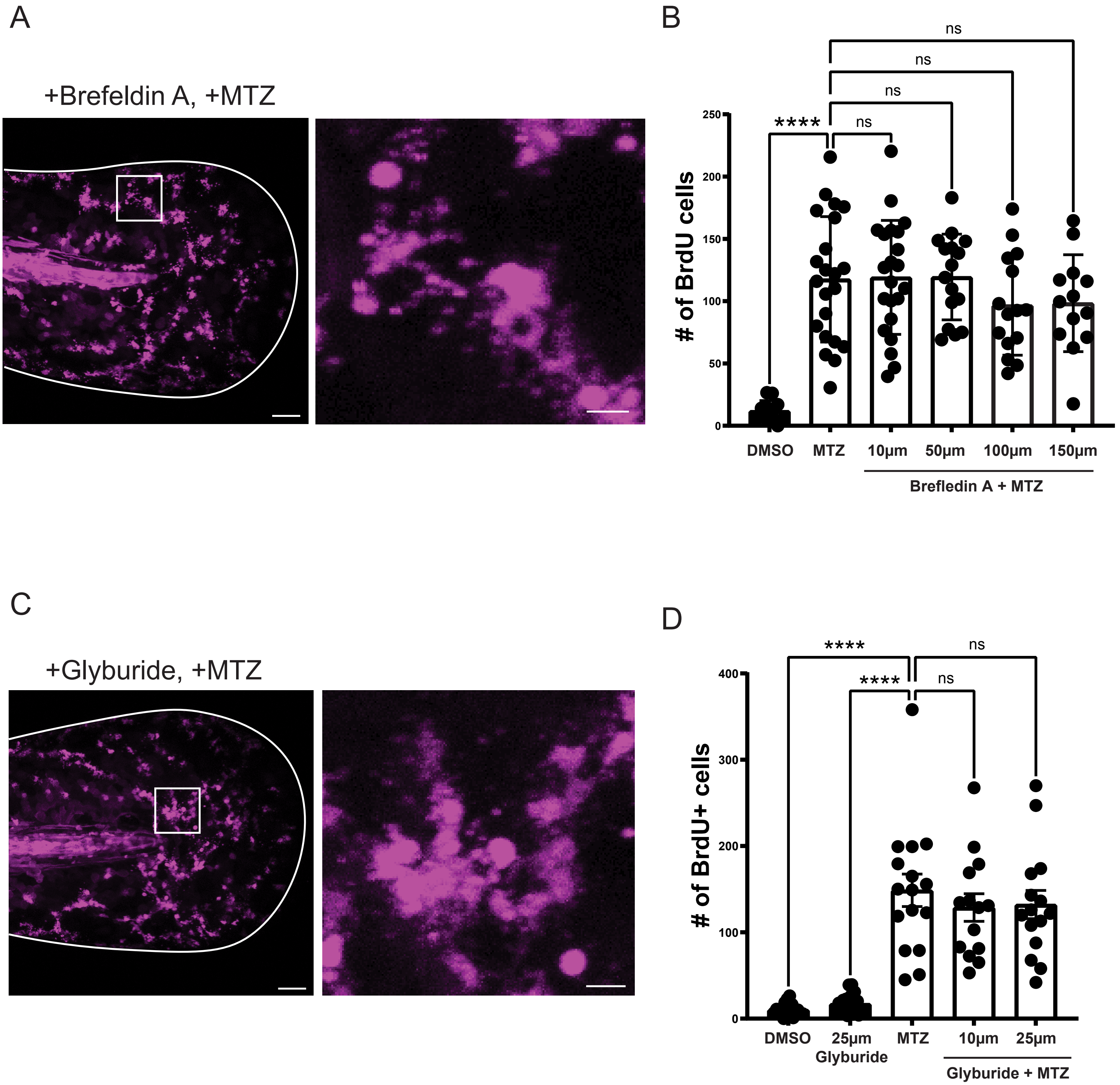

Supplement: S6 Fig — (A) Treatment with Brefeldin A does not affect AEV formation. (B) Treatment with increasing concentrations of Brefeldin A does not induce a significant reduction in proliferation. (C) esAEV formation after treatment with glyburide. (D) Apoptosis-induced proliferation in the presence of glyburide. Each condition had 32–40 larvae. Statistical significance was calculated with a two-way ANOVA with a Tukey’s post hoc test. Scales bars = 50 μm. The underlying data for the graphs in this figure can be found in S2 Data. (PDF) [file pbio.3002194.s006.pdf]
